# Supplementary material for: Risk factors for third-generation cephalosporin-resistant and extended-spectrum β-lactamase-producing Escherichia coli carriage in domestic animals of semirural parishes east of Quito, Ecuador
Source: PLOS Glob Public Health. 2022 Mar 23;2(3):e0000206. doi: 10.1371/journal.pgph.0000206 (PMC10021719; doi:10.1371/journal.pgph.0000206)
Supplement: S1 Survey — (PDF) [file pgph.0000206.s001.pdf]

Universidad San Francisco

Participante ID:

CUESTIONARIO
ESTUDIO PARA LA PREVENCIÓN DE RESISTENCIA A ANTIBIÓTICOS EN LA COMUNIDAD

(Leer consentimiento informado. Si el responsable del menor cumple con los criterios de inscripción, acepta participar en el estudio y firma el consentimiento informado, continúe con la encuesta.)

1.	Nombre del encuestador								
2.	Nombre del barrio								
3.	Casa ID								
4.	Visita Número (Ciclo)								
5.	Fecha de la encuesta	D	D	M	M	A	A	A	A
6.	Coordinadas del GPS colectados								

Sección A: Información del encuestado

Nº.	PREGUNTA	RESPUESTA	CÓDIGO	SALTO
7.	¿Cuál es su primer nombre?	Nombre: ___________________________<br>No responde: 999		___
8.	¿Cuántos años tiene (años cumplidos)?	Años: __________<br>No responde: 999		___
9.	Género	Masculino: 1<br>Femenino: 2<br>Transgénero: 3<br>No responde: 999		___
10.	Grupo étnico:	Afro-ecuatoriano: 1<br>Mestizo: 2<br>Mulato: 3<br>Blanco: 4<br>Indígena: 5<br>Otro: 6<br>No sabe: 96<br>No responde: 999		___
11.	¿Cuántas personas viven actualmente en su casa?	# de miembros: ____________________<br>No responde: 999		___
12.	Entre ellos, ¿cuántos son niños menores de 12 años?	# de niños (0-12 años): __________<br>No responde: 999		___

Sección B: Estatus socioeconómico del hogar

Nº.	PREGUNTA	RESPUESTA	CÓDIGO	SALTO
13.	¿Puede solo leer, solo escribir, leer y escribir, o no sabe ni leer ni escribir?	Leer y escribir: 1<br>Solo leer: 2<br>No lee ni escribe: 3<br>No responde: 999		Si la respuesta es "3" o "999" vaya a #16
14.	¿Usted alguna vez asistió a la escuela?	Sí: 1<br>No: 0<br>No responde: 999		Si la respuesta es "0" o "999" vaya a #16
15.	¿Cuál es su nivel de educación más alto?	Primaria: 1<br>Secundaria: 2<br>Superior: 3<br>No responde: 999		—
16.	¿Usted o algún miembro de su hogar posee alguna de las siguientes cosas?<br><br>*Marque todas las opciones aplicables*	Televisión que funcione: ☐<br>Direct TV o Televisión Satelital: ☐<br>Un carro o camioneta que sirva: ☐<br>Más de un automóvil: ☐<br>Computadora: ☐<br>Internet: ☐<br>Esta casa: ☐<br>Estas tierras/propiedad: ☐<br>No responde: ☐		—

Sección C: Condición ambiental de salud del hogar

Nº.	PREGUNTA	RESPUESTA	CÓDIGO	SALTO
17.	¿Cuál es la principal fuente de agua para beber en su casa?<br>*Marque solamente una opción*	Agua potable dentro de la casa: 1<br>Agua potable en el patio: 2<br>Llave pública: 3<br>Pozo/manantial protegido: 4<br>Agua superficial: 5<br>Agua embotellada: 6<br>Otra: 7<br>No responde: 999		—
18.	¿Con qué frecuencia tiene disponible agua para beber?	Todo el día (24 horas): 1<br>Más de una vez por día: 2<br>Una vez por día: 3<br>Una vez a cada dos días: 4<br>Una vez a cada tres días: 5<br>Otra: 6<br>No responde: 999		—
19.	¿Trata usted el agua para beber de alguna manera?	Sí: 1<br>No: 2<br>No sabe: 96<br>No responde: 999		Si la respuesta es “2”,”96” o “999” vaya a #21
20.	¿Qué suele hacer principalmente para que el agua sea segura para beber?<br>*Marque solamente una opción*	Hierve: 1<br>Pone legía/Cloro: 2<br>Filtra el agua (cerámica/arena/compuesto/etc.): 3<br>Cierne en un paño: 4<br>Deja que se asiente: 5<br>Otra: 6<br>No sabe: 96<br>No responde: 999		—
21.	¿Qué tipo de instalación sanitaria usa su familia generalmente?<br>*Marque solamente una opción*	Inodoro que va al alcantarillado: 1<br>Inodoro con pozo séptico: 2<br>Inodoro con cisterna: 3<br>Inodoro, pero no sabe a dónde se dirige: 4<br>Letrina con piso duro y ventilación: 5<br>Letrina sin piso duro: 6<br>En el campo libre: 7<br>Otro: 8<br>No sabe: 96<br>No responde: 999		—
22.	¿Hay agua y jabón disponibles en este momento en el lugar donde usted lava las manos con más frecuencia?	Agua y jabón disponibles: 1<br>Solo jabón: 2<br>Solo agua: 3<br>Ni agua ni jabón disponibles: 4<br>No responde: 999		—

23.	¿Cuántas habitaciones en esta casa se utilizan para dormir?	Número de habitaciones: ____<br>No responde: 999		——
24.	¿Por lo general, asiste a la guardería/escuela su niño/a participante del estudio?	Si: 1<br>No: 0<br>No sabe: 96<br>No responde: 999		Si la respuesta es "0", "96" o "999" vaya a #26
25.	¿En las últimas 2 semanas, con qué frecuencia ha asistido a la guardería su hijo/a?	Menos de una vez: 1<br>1 a 3 veces: 2<br>4 a 6 veces: 3<br>7 veces o más: 4<br>No sabe: 96<br>No responde: 999		——
26.	¿Usted siente olor a gallinero, ganado o chancho cerca de su casa?	Nunca: 1<br>Menos de una vez en la semana: 2<br>1 a 3 veces en la semana: 3<br>4 a 6 veces en la semana: 4<br>7 veces o más: 5<br>No sabe: 96<br>No responde: 999		——
27.	¿Usted tiene conocimiento de algún criadero de pollos (con más de 500 pollos) o chanchera que este cerca de su casa?	Si: 1<br>No: 0<br>No sabe: 96<br>No responde: 999		Si la respuesta es "0", "96" o "999" vaya a #29
28.	En caso afirmativo, ¿a qué distancia está de su casa?	_______________ metros		——
29.	La última vez que su hijo/a defecó, ¿qué hizo usted para deshacerse de las heces?	Usó el inodoro/letrina: 1<br>Lavo o puso dentro del inodoro/letrina: 2<br>Lavo o puso en el drenaje o la zanja: 3<br>Desechó en la basura: 4<br>Enterró: 5<br>Lo dejó al aire libre: 6<br>Otro: 7<br>No sabe: 96<br>No responde: 999		——

Sección D: Uso de Antibióticos en el hogar

Nº.	PREGUNTA	RESPUESTA	CÓDIGO	SALTO
30.	¿En las últimas 2 semanas, usted ha tenido en su casa alguien que haya sufrido de alguna infección o enfermedad?	Sí: 1<br>No: 0<br>No sabe/No recuerda: 96<br>No responde: 999		Si la respuesta es "0", "96" o "999" vaya a #37
31.	¿Este miembro del hogar que estuvo enfermo, recibió atención médica para esta enfermedad?	Sí: 1<br>No: 0<br>No sabe/No recuerda: 96<br>No responde: 999		Si la respuesta es "0", "96" o "999" vaya a #33
32.	En caso afirmativo, ¿dónde recibió el miembro enfermo del hogar los cuidados de salud?	Hospital: 1<br>Farmacia: 2<br>Clínica privada: 3<br>Otro: 4<br>No sabe/No recuerda: 96<br>No responde: 999		—
33.	¿El miembro enfermo del hogar tomó antibiótico para esta enfermedad?	Sí: 1<br>No: 0<br>No sabe/No recuerda: 96<br>No responde: 999		Si la respuesta es "0", "96" o "999" vaya a #37
34.	¿En caso afirmativo, sabe cuál fue el nombre del antibiótico(s)?	Si: 1<br>No: 0<br>No sabe/No recuerda: 96<br>No responde: 999		Si la respuesta es "0", "96" o "999" vaya a #36
35.	¿Cuál fue el nombre del antibiótico(s) usado?	Nombre (s): ____________________		—
36.	¿Por cuantos días el miembro enfermo del hogar tomó antibiótico?	1 día: 1<br>2 días: 2<br>3 días: 3<br>Más de 3 días: 4<br>No responde: 999		—
37.	¿En los últimos 3 meses, usted ha tenido en su casa alguien que haya sufrido alguna infección o enfermedad?	Sí: 1<br>No: 0<br>No sabe/No recuerda: 96<br>No responde: 999		Si la respuesta es "0", "96" o "999" vaya a #42
38.	¿El miembro enfermo del hogar tomó antibióticos para esta enfermedad?	Si: 1<br>No: 0<br>No sabe/No recuerda: 96<br>No responde: 999		Si la respuesta es "0", "96" o "999" vaya a #42

39.	¿En caso afirmativo, usted sabe el nombre del antibiótico(s)?	Si: 1<br>No: 0<br>No responde: 999		Si la respuesta es "0" o "999" vaya a #41
40.	¿Cuál fue el nombre del antibiótico(s)?	Nombre de antibiótico(s): ____________________		——
41.	¿Por cuántos días tomó el antibiótico(s)?	1 día: 1<br>2 días: 2<br>3 días: 3<br>Más de 3 días: 4<br>No sabe/No recuerda: 96<br>No responde: 999		——

Sección E: Salud y actividades sanitarias de los niños pequeños

Nº.	PREGUNTA	RESPUESTA	CÓDIGO	SALTO
42.	¿Cuál es el primer nombre de su hijo/a participante del proyecto?	Nombre: ______________________________<br>No responde: 999		—
43.	¿Qué edad tiene su hijo/a?<br>[*Esto identifica al niño que proveerá la muestra fecal*]	Edad: ______________________________<br>*Usar edad de 0.3 (3 meses) hasta 5.00 años*		—
44.	¿Cuál es la fecha de nacimiento de su hijo/a?	*Día/Mes/Año*<br>*Ejemplo: 31/01/2001*		—
45.	Género	Masculino: 1<br>Femenino: 2<br>No responde: 999		—
46.	¿Su hijo/a todavía toma leche materna?	A veces: 1<br>Siempre: 2<br>No: 3<br>No responde: 999		—
47.	¿En los últimos 3 meses, su hijo/a ha recibió algún tratamiento médico?	Sí: 1<br>No: 0<br>No sabe/No recuerda: 96<br>No responde: 999		Si la respuesta es “0”, “96” o “999” vaya a #49
48.	¿Dónde recibió el miembro enfermo del hogar el cuidado de salud?	Hospital: 1<br>Farmacia: 2<br>Clínica privada: 3<br>Otro: 4<br>No sabe/No recuerda: 96<br>No responde: 999		—
49.	¿En los últimos 3 meses, su hijo/a ha tomado antibiótico para alguna enfermedad o infección?	Sí: 1<br>No: 0<br>No sabe/No recuerda: 96<br>No responde: 999		Si la respuesta es “0”, “96” o “999” vaya a #54
50.	¿En caso afirmativo, por cuántos días?	Número de días: ________<br>No responde: 999		—
51.	¿Usted sabe el nombre del antibiótico usado?	Sí: 1<br>No: 0<br>No recuerda: 96<br>No responde: 999		Si la respuesta es “0”, “96” o “999” vaya a #53
52.	¿Cuál fue el nombre del antibiótico(s) usado(s)?	Antibiótico(s): ________________________		—

53.	¿Es posible ver la caja/botella del antibiótico o la receta?<br>*Sacar una foto con el Tablet*	Sí: 1<br>No: 0<br>No lo encuentra: 96<br>No responde: 999		___
54.	¿A dónde va usted en general a comprar antibióticos?	Hospital: 1<br>Centro de salud: 2<br>Farmacia: 3<br>De un familiar o amigo: 4<br>Otra localización: 5<br>No sabe/No recuerda: 96<br>No responde: 999		___
55.	¿En los últimos 3 meses, usted ha administrado antiparasitario a su hijo/a?	Sí: 1<br>No: 0<br>No sabe/No recuerda: 96<br>No responde: 999		___
56.	¿En los últimos 3 meses, su hijo/a ha tomado algún otro medicamento o vitaminas por una enfermedad o alguna otra razón?	Sí: 1<br>No: 0<br>No sabe/No recuerda: 3<br>No responde: 999		___
57.	¿En los pasados 3 meses, su hijo/a ha tenido contacto con ganado, cerdos o aves de corral?	Menos de una vez en la semana: 1<br>1 a 2 veces en la semana: 2<br>3 veces o más en la semana: 3<br>No: 4<br>No sabe: 96<br>No responde: 999		___
58.	¿En los pasados 3 meses, su hijo/a ha tenido contacto con mascotas (por ejemplo, perros o gatos)?	Menos de una vez en la semana: 1<br>1 a 2 veces en la semana: 2<br>3 veces o más en la semana: 3<br>No: 4<br>No sabe: 96<br>No responde: 999		___
59.	¿Cuándo su hijo/a tiene contacto con animales, se lava las manos, o ustedes le lavan las manos?	Siempre: 1<br>A veces: 2<br>Raramente: 3<br>Nunca: 4<br>No sabe: 96<br>No responde: 999		___
60.	¿En las últimas 2 semanas, su hijo/a ha consumido leche, queso, huevos, carne de animales o aves de corral producidos/criados en su hogar?	Menos de una vez en la semana: 1<br>1 a 2 veces en la semana: 2<br>3 veces o más en la semana: 3<br>No: 4<br>No sabe: 96<br>No responde: 999		___

61.	¿En las últimas 2 semanas, su hijo/a ha consumido carne, queso, huevos o aves de corral compradas fuera de su hogar?	Menos de una vez en la semana: 1<br>1 a 2 veces en la semana: 2<br>3 veces o más en la semana: 3<br>No: 4<br>No sabe: 96<br>No responde: 999		___
62.	¿En los últimos 7 días su hijo/a ha presentado diarrea ("considerándose esto como 3 o más deposiciones sueltas o líquidas por día")?	Sí: 1<br>No: 0<br>No sabe: 96<br>No responde: 999		___
63.	¿Ha tenido su hijo/a alguna erupción con picazón durante los últimos 3 meses?	Sí: 1<br>No: 0<br>No sabe: 96<br>No responde: 999		___
64	¿Algún médico ha diagnosticado a su hijo/a con asma?	Sí: 1<br>No: 0<br>No sabe: 96<br>No responde: 999		___

UNIVERSIDAD SAN FRANCISCO

Sección F: Actividades de higiene de los miembros del hogar

Nº.	PREGUNTA	RESPUESTA	CÓDIGO	SALTO
65.	¿Durante los últimos 6 meses, alguien de su hogar visitó o trabajó en un hospital o clínica?	Menos de una vez en la semana: 1<br>1 a 2 veces en la semana: 2<br>3 veces o más en la semana: 3<br>No: 4<br>No sabe: 96<br>No responde: 999		—
66.	¿Durante los últimos 6 meses, alguien de su hogar trabaja fuera del hogar con animales?	Menos de una vez en la semana: 1<br>1 a 2 veces en la semana: 2<br>3 veces o más en la semana: 3<br>No: 4<br>No sabe: 96<br>No responde: 999		Si la respuesta es "4", "96" o "999" vaya a #68
67.	¿Con qué animales trabajó?	Nombre de animal(es): ________________________<br>No sabe/No recuerda: 96<br>No responde: 999		—
68.	¿Durante los últimos 6 meses, alguien de su hogar ha sacrificado ganado, o gallinas, o ha trabajado en el proceso de animales o productos derivados como carne, aves, queso o leche, para el consumo o la venta?	Menos de una vez en la semana: 1<br>1 a 2 veces en la semana: 2<br>3 veces o más en la semana: 3<br>No: 4<br>No sabe: 96<br>No responde: 999		—
69.	¿Durante los últimos 6 meses, alguien manipuló, trató o desechó heces humanas o heces animales que no sean los de su hogar?	Menos de una vez en la semana: 1<br>1 a 2 veces en la semana: 2<br>3 veces o más en la semana: 3<br>No: 4<br>No sabe: 96<br>No responde: 999		—

Sección G: Conocimiento, actitudes y prácticas (CAP) acerca de la resistencia a antibióticos

Nº.	PREGUNTA	RESPUESTA	CÓDIGO	SALTO
70.	Cuando le duele la garganta a su hijo/a, ¿le da antibióticos?	Siempre: 1<br>A veces: 2<br>Nunca: 3<br>No sabe: 96<br>No responde: 999		—
71.	Cuando su hijo/a se resfría, los antibióticos los ayudarán a mejorar más rápidamente?	Siempre: 1<br>A veces: 2<br>No: 3<br>No sabe: 96<br>No responde: 999		—
72.	Cuando su hijo/a tiene un resfriado fuerte, como para ir a un médico, ¿usted espera que le médico le recete antibióticos?	Siempre: 1<br>A veces: 2<br>Nunca: 3<br>No sabe: 96<br>No responde: 999		—
73.	¿Está bien usar antibióticos cuando se siente enfermo, para ayudar a curarse?	Siempre: 1<br>A veces: 2<br>Nunca: 3<br>No sabe: 96<br>No responde: 999		—
74.	Si su hijo/a tiene bronquitis y no puede llevarlo al médico rápidamente, ¿es una opción para usted ir una farmacia para comprar antibióticos?	Siempre: 1<br>A veces: 2<br>Nunca: 3<br>No sabe: 96<br>No responde: 999		—
75.	¿La mayoría de sus amigos piensan que deben dar antibióticos a sus hijos cuando tienen un resfriado?	Siempre: 1<br>A veces: 2<br>Nunca: 3<br>No sabe: 96<br>No responde: 999		—
76.	¿La mayoría de sus amigos piensan que deben dar antibióticos a sus hijos cuando tienen una diarrea?	Siempre: 1<br>A veces: 2<br>Nunca: 3<br>No sabe: 96<br>No responde: 999		—
77.	¿La mayoría de sus amigos piensan que deben dar antibióticos a sus hijos cuando tienen erupciones en la piel?	Siempre: 1<br>A veces: 2<br>Nunca: 3<br>No sabe: 96<br>No responde: 999		—

78.	¿La mayoría de sus amigos compran antibióticos para sus hijos enfermos sin receta médica?	Siempre: 1<br>A veces: 2<br>Nunca: 3<br>No sabe: 96<br>No responde: 999		—
79.	¿Los antibióticos pueden matar bacterias?	Sí: 1<br>No: 0<br>No sabe: 96<br>No responde: 999		—
80.	¿Los antibióticos pueden matar virus?	Sí: 1<br>No: 0<br>No sabe: 96<br>No responde: 999		—

Sección H: Información de mascotas, ganado y aves de corral

Nº.	PREGUNTA	RESPUESTA	CÓDIGO	SALTO
81.	¿En las últimas 3 semanas, su hijo/a ha tenido contacto con mascotas domésticas, ganado o aves de corral?	Menos de una vez en la semana: 1<br>1 a 2 veces en la semana: 2<br>3 veces o más en la semana: 3<br>No: 4<br>No sabe: 96<br>No responde: 999		—
82.	¿Actualmente usted tiene algún animal en su propiedad?	Sí: 1<br>No: 0<br>No responde: 999		Si la respuesta es "0" o "999" se acabó
83.	¿Cuantos pollos usted tiene en su propiedad?			—
84.	¿Cuantos cuyes usted tiene en su propiedad?			—
85.	¿Cuantos cerdos usted tiene en su propiedad?			—
86.	¿Cuántas vacas / ganados usted tiene en su propiedad?			—
87.	¿Cuantos perros usted tiene en su propiedad?			—
88.	¿Cuantos patos / gansos usted tiene en su propiedad?			—
89.	¿Cuantas ovejas / borregos usted tiene en su propiedad?			—
90.	¿Cuantos conejos usted tiene en su propiedad?			—
91.	¿Cuantas cabras usted tiene en su propiedad?			—
92.	¿Cuantos gatos usted tiene en su propiedad?			—
93	¿Cuánta codorniz usted tiene en su propiedad?			—
94.	¿Otro animal en su propiedad? ¿Cuantos?			—
**Nº.**	**PREGUNTA**	**RESPUESTA**	**CÓDIGO**	**SALTO**
95.	¿Usted permite que mascotas u otros animales entren a su casa?	Menos de una vez en la semana: 1<br>1 a 2 veces en la semana: 2<br>3 veces o más en la semana: 3<br>Nunca: 4<br>No sabe: 96<br>No responde: 999		—
96.	¿En las últimas 3 semanas, algún animal ingresó en un área donde su hijo/a pasa su tiempo?	Menos de una vez en la semana: 1<br>1 a 2 veces en la semana: 2<br>3 veces o más en la semana: 3<br>No: 4<br>No sabe: 96<br>No responde: 999		—
97.	¿En las últimas 3 semanas, su hijo/a jugó en áreas donde los animales defecan?	Menos de una vez en la semana: 1<br>1 a 2 veces en la semana: 2<br>3 veces o más en la semana: 3<br>No: 4		—

		No sabe: 96<br>No responde: 999		
98.	¿Cómo maneja usted los desechos de animales en su jardín/patio/terreno?	Lo deja en el patio para que se descomponga o lo descompone:1<br>Lo almacena y luego lo coloca en su terreno: 2<br>Lo usa en los cultivos como fertilizante: 3<br>Véndelo: 4<br>Bota en la basura: 5<br>No aplica: 6<br>Otro: 7<br>No sabe: 96<br>No responde: 999		—
99.	¿En las últimas 3 semanas, su ganado y/o aves de corral consumieron agua del rio o acequia?	Sí: 1<br>No: 2<br>No aplica: 3<br>No sabe/No recuerda: 96<br>No responde: 999		—
100.	¿En los últimos 6 meses, usted ha administrado antibióticos a sus animales, ganado o pollos?	Sí: 1<br>No: 0<br>No sabe/No recuerda: 96<br>No responde: 999		Si la respuesta es "0", "96" o "999" vaya a #106
101.	¿Dónde compra u obtiene los antibióticos que usa con sus animales?	Veterinario: 1<br>Tienda de alimentos para animales: 2<br>Farmacia: 3<br>De un familiar o amigo: 4<br>Otra localización: 5<br>No sabe/No recuerda: 96<br>No responde: 999		—
102.	¿Cuáles instrucciones recibió usted sobre el uso de antibióticos en animales?	Instrucciones escritas: 1<br>Instrucciones orales: 2<br>No recibe instrucciones: 3<br>Otro profesional administra: 4<br>No sabe/No recuerda: 96<br>No responde: 999		—
103.	¿A qué animales usted ha administrado antibióticos?	Pollo: ☐<br>Cuy: ☐<br>Cerdo: ☐<br>Vacas/ganado: ☐<br>Perros: ☐<br>Patos/gansos: ☐<br>Ovejas/borregos: ☐<br>Conejos: ☐<br>Cabras: ☐<br>Gatos: ☐<br>Codorniz: ☐<br>Otros animales:☐		—

UNIVERSIDAD SAN FRANCISCO

104.	¿Con qué frecuencia administra antibióticos al ganado o aves de corral u otros animales?	Según sea necesario: 1<br>Con alguna frecuencia especifica: 2<br>Todos los días: 3<br>No sabe/no recuerda: 96<br>No responde: 999		—
105.	¿Cuáles son sus razones para usarlos en los animales (los antibióticos)?<br><br>*Marcar todo lo necesario*	Aumenta el crecimiento de los animales: ☐<br>Evite que los animales se enfermen: ☐<br>Solo da cuando animales están enfermos: ☐<br>Fue recomendado por un veterinario: ☐<br>Fue recomendado por un farmacéutico: ☐<br>Otro: ☐<br>No sabe: ☐<br>No responde: ☐		—
106.	¿Es posible ver el empaque del antibiótico(s)?<br>*Sacar una foto con el Tablet*	Sí: 1<br>No: 0<br>No lo encuentra: 3<br>No responde: 999		—
107.	¿En los últimos 6 meses, usted ha administrado alguna otra medicina o vitaminas a sus animales, ganado o pollos?	Sí: 1<br>No: 0<br>No sabe/No recuerda: 96<br>No responde: 999		—
108.	¿Usted da balanceados comerciales para alimentar a sus animales (por ejemplo, ganado, cerdos o pollos)?	Sí: 1<br>No: 0<br>No sabe: 96<br>No responde: 999		Si la respuesta es "0", "96" o "999" **se acabó**
109.	En caso afirmativo, ¿cuál es el nombre?	Nombre: ____________________________<br>No sabe: 96<br>No responde: 999		—
110.	¿Es posible ver el empaque?<br><br>*Sacar una foto con el Tablet*	Sí: 1<br>No: 0<br>No lo encuentra: 2<br>No responde: 999		—
