# Supplementary material for: Risk factors for third-generation cephalosporin-resistant and extended-spectrum β-lactamase-producing Escherichia coli carriage in domestic animals of semirural parishes east of Quito, Ecuador
Source: PLOS Glob Public Health. 2022 Mar 23;2(3):e0000206. doi: 10.1371/journal.pgph.0000206 (PMC10021719; doi:10.1371/journal.pgph.0000206)
Supplement: S2 Survey — (PDF) [file pgph.0000206.s002.pdf]

UNIVERSIDAD SAN FRANCISCO

Participant ID:

QUESTIONNAIRE

STUDY FOR THE PREVENTION OF ANTIBIOTIC RESISTANCE IN THE COMMUNITY

(Read informed consent. If the primary caregiver meets the enrollment criteria, agrees to participate in the study, and has signed the informed consent, continue with the survey.)

1.	Investigator First Name								
2.	Neighborhood Name								
3.	Household ID								
4.	Visit Number (Cycle)								
5.	Date of survey:	D	D	M	M	Y	Y	Y	Y
6.	GPS Coordinates								

Section A: Information about the respondent

Nº.	QUESTION	RESPONSE	CODE	JUMP
7.	What is your first name?	Name: ________________<br>No response: 999		—
8.	How old are you (in completed years)?<br>(Enter 999 for 'Does not respond')	Age: _________<br>No response: 999		—
9.	Sex:	Male: 1<br>Female: 2<br>Transgender: 3<br>No response: 999		—
10.	Ethnic group:	Afro-Ecuatorian: 1<br>Mestizo: 2<br>Mulatto: 3<br>White: 4<br>Indigenous: 5<br>Others: 6<br>Don't know: 96<br>No response: 999		—
11.	How many people currently live in your home?	# of members: ____________<br>No response: 999		—
12.	Among the people currently living in your home, how many are children aged less than 12 years?	# of children (0-12 years): _____<br>No response: 999		—

Section B: Household socioeconomic status

Nº.	QUESTION	RESPONSE	CODE	JUMP
13.	Can you only read, only write, read and write, or do you not know how to read nor write?	Read and write: 1<br>Only read: 2<br>Neither read nor write: 3<br>No response: 999		If "3" or "999" go to #16
14.	Have you ever attended school?	Yes: 1<br>No: 0<br>No response: 999		If "0" or "999", go to #16
15.	What is your highest level of education?	Primary: 1<br>High school: 2<br>University: 3<br>No response: 999		—
16.	Do you or any member of your household own any of the following items?<br>*Select all that apply*	Working television: ☐<br>Direct TV or satellite television: ☐<br>Working car or truck: ☐<br>More than one car: ☐<br>Computer: ☐<br>Internet: ☐<br>This House: ☐<br>This lands/property: ☐<br>No response: ☐		—

UNIVERSIDAD SAN FRANCISCO

Section C: Household environmental health conditions

Nº.	QUESTION	RESPONSE	CODE	JUMP
17.	What is the main source of drinking water at home?<br><br>*Select only one*	Drinking water from inside the house: 1<br>Drinking water from the patio: 2<br>Public faucet: 3<br>Well or protected spring: 4<br>Surface water: 5<br>Bottled water: 6<br>Other: 7<br>No response: 999		—
18.	How often do you have drinking water available?	All day (24-hour supply): 1<br>Available more than once a day: 2<br>Available once a day: 3<br>Once every two days: 4<br>Once every three days: 5<br>Other: 6<br>No response: 999		—
19.	Do you treat the water to make it safe to drink?	Yes: 1<br>No: 2<br>Don't know: 96<br>No response: 999		If "2", "96" or "999" go to #22
20.	What do you mainly do to make the water safe to drink?<br><br>*Select only one*	Boil it: 1<br>Put in lime/chlorine: 2<br>Filter the water (ceramic/sand/compound/etc.): 3<br>Sieve in a cloth: 4<br>Leave it to settle: 5<br>Other: 6<br>Don't know: 96<br>No response: 999		—
21.	What type of sanitary system is most often used by your family?<br><br>*Select only one*	Toilet that goes into the sewer: 1<br>Septic toilet: 2<br>Toilet with cistern: 3<br>Toilet, but you don't know where it goes: 4<br>Latrine with hard floor and ventilation: 5<br>Latrine without hard floor: 6<br>Outside: 7<br>Other: 8<br>Don't know: 96<br>No response: 999		—
22.	Are water and soap currently available at the place where you most often wash your hands?	Water and soap are available: 1<br>Only soap: 2<br>Only water: 3<br>Neither water nor soap available: 4<br>No response: 999		—

23.	How many rooms in this House are used for sleeping?	Number of rooms: ____<br>No response: 999		—
24.	In general, does your child participating in this study attend nursery school/school?	Yes: 1<br>No: 0<br>Don't know: 96<br>No response: 999		If "0","96" or "999" go to #26
25.	In the last 2 weeks, how often has your child attended nursery school?	Less than once: 1<br>1 to 3 times: 2<br>4 to 6 times: 3<br>7 times or more: 4<br>Other: 5<br>Don't know: 96<br>No response: 999		—
26.	Do you smell odors from commercial poultry, cattle, or swine farms near your home?	Never: 0<br>Less than 1 time per week: 1<br>1 to 3 times per week: 2<br>4 to 6 times per week: 3<br>More than 7 times per week: 4<br>Other: 5<br>Don't know: 96<br>No response: 999		—
27.	Do you have knowledge of any poultry farms (with more than 500 chickens) or swine farms near your home?	Yes: 1<br>No: 0<br>Don't know: 96<br>No response: 999		If "0","96" or "999" go to #29
28.	If so, how far is the farm from your home?	_____________ meters		—
29.	The last time that your child defecated, how did you dispose of their feces?	They used the toilet/latrine: 1<br>You cleaned or placed it in the toilet/latrine: 2<br>You cleaned or placed it in the drain or ditch: 3<br>You threw it away in the trash: 4<br>You buried it: 5<br>You left it outside: 6<br>Other: 7<br>Don't know: 96<br>No response: 999		—

Section D: Household use of antibiotics

Nº.	QUESTION	RESPONSE	CODE	JUMP
30.	In the last 2 weeks, has anyone in your home suffered from any infection or illness?	Yes: 1<br>No: 0<br>Don't know/Don't remember: 96<br>No response: 999		If "0", "96" or "999" go to #37
31.	Did this member of the household who was sick receive medical attention for this illness?	Yes: 1<br>No: 0<br>Don't know/Don't remember: 96<br>No response: 999		If "0", "96" or "999" go to #33
32.	If so, where did the sick household member receive healthcare?	Hospital: 1<br>Pharmacy: 2<br>Private clinic: 3<br>Other: 4<br>Don't know/Don't remember: 96<br>No response: 999		——
33.	Did the sick household member take antibiotics for this illness?	Yes: 1<br>No: 0<br>Don't know/Don't remember: 96<br>No response: 999		If "0", "96" or "999" go to #37
34.	If so, do you know the name of the antibiotic(s) used?	Yes: 1<br>No: 0<br>Don't know/Don't remember: 3<br>No response: 999		If "0","96" or "999" go to #39
35.	What was the name of the antibiotic(s) used?	Name(s): ___________________		——
36.	For how many days did the household member take antibiotics?	1 day: 1<br>2 days: 2<br>3 days: 3<br>More than 3 days: 4<br>No response: 999		——
37.	In the last 3 months, has anyone in your home suffered from any infection or illness?	Yes: 1<br>No: 0<br>Don't know/Don't remember: 96<br>No response: 999		If "0", "96" or "999" go to #42
38.	Did the sick household member take antibiotics for this condition?	Yes: 1<br>No: 0<br>Don't know/Don't remember: 96<br>No response: 999		If "0", "96" or "999" go to #42
39.	If so, do you know the names of the antibiotic(s)?	Yes: 1<br>No: 0<br>No response: 999		If "0" or "999" go to #41

40.	What was the name of the antibiotic(s)?	Name of the antibiotic(s): _______		___
41.	For how many days did they take the antibiotic(s)?	1 day: 1<br>2 days: 2<br>3 days: 3<br>More than 3 days: 4<br>Don't know/Don't remember: 96<br>No response: 999		___

Section E: Health and hygiene behaviors of young children

Nº.	QUESTION	RESPONSE	CODE	JUMP
42.	What is the first name of your child participating in this study?	Name: ____________________<br>No response: 999		___
43.	How old is your child?<br>[*This identifies the child who will provide the fecal sample*]	Age: ______________________<br>*Use ages from 0.3 (3 months) to 5 years*		___
44.	What is your child's birthday?	*Day/Month/Year*<br>*Example: 31/01/2001*		___
45.	Gender	Male: 1<br>Female: 2<br>No response: 999		___
46.	Does your child still breastfeed?	Sometimes: 1<br>Always: 2<br>No: 3<br>No response: 999		___
47.	In the last 3 months, has your child received any medical treatment?	Yes: 1<br>No: 0<br>Don't know/Don't remember: 96<br>No response: 999		If "0", "96" or "999" go to #49
48.	Where did the sick household member receive healthcare?	Hospital: 1<br>Pharmacy: 2<br>Private clinic: 3<br>Other: 4<br>Don't know/Don't remember: 96<br>No response: 999		___
49.	In the last 3 months, has your child taken antibiotics for any illness or infection?	Yes: 1<br>No: 0<br>Don't know/Don't remember: 3<br>No response: 999		If "0", "96" or "999" go to #54
50.	If so, for how many days?	Number of days: ________<br>No response: 999		___
51.	Do you know the name of the antibiotic used?	Yes: 1<br>No: 0<br>Don't remember: 3<br>No response: 999		If "0", "96" or "999" go to #53
52.	What was the name of the antibiotic(s) used?	Antibiotic(s): ________________		___
53.	Is it possible to see the antibiotic container/bottle or the prescription?<br>*Take a picture with the Tablet*	Yes: 1<br>No: 0<br>Could not find: 96<br>No response: 999		___

54.	Where do you generally go to buy antibiotics?	Hospital: 1<br>Health Center: 2<br>Pharmacy: 3<br>A family member or friend: 4<br>Other location: 5<br>Don't know/Don't remember: 96<br>No response: 999		___
55.	In the last 3 months, have you given your child an antiparasitic?	Yes: 1<br>No: 0<br>Don't know/Don't remember: 96<br>No response: 999		___
56.	In the last 3 months, has your child taken any other medications or vitamins for an illness or other reason?	Yes: 1<br>No: 0<br>Don't know/Don't remember: 96<br>No response: 999		___
57.	In the last 3 months, has your child had contact with cattle, swine or poultry?	Less than once per week: 1<br>1 to 2 times per week: 2<br>3 or more times per week: 3<br>No: 4<br>Don't know: 96<br>No response: 999		___
58.	In the past 3 months, has your child had contact with pets (for example, dogs or cats)?	Less than once per week: 1<br>1 to 2 times per week: 2<br>3 or more times per week: 3<br>No: 4<br>Don't know: 96<br>No response: 999		___
59.	When your child has contact with animals, do they wash their hands, or do you wash their hands?	Always: 1<br>Sometimes: 2<br>Rarely: 3<br>Never: 4<br>Don't know: 96<br>No response: 999		___
60.	In the last 2 weeks, has your child consumed milk, cheese, eggs, animal meat or poultry produced/raised at your household?	Less than once per week: 1<br>1 to 2 times per week: 2<br>3 or more times per week: 3<br>No: 4<br>Don't know: 96<br>No response: 999		___
61.	In the last 2 weeks, has your child eaten meat, cheese, eggs or poultry purchased outside of your home?	Less than once per week: 1<br>1 to 2 times per week: 2<br>3 or more times per week: 3<br>No: 4<br>Don't know: 96<br>No response: 999		___

62.	In the last 7 days, has your child had diarrhea ("defined as 3 or more loose or liquid stools in 1 day")?	Yes: 1<br>No: 2<br>Don't know: 96<br>No response: 999		___
63.	Has your youngest child had any rashes with itching in the last 3 months?	Yes: 1<br>No: 2<br>Don't know: 96<br>No response: 999		___
64.	Has a doctor every diagnosed your child with asthma?	Yes: 1<br>No: 2<br>Don't know: 3<br>No response: 999		___

Section F: Hygiene behaviors of household members

Nº.	QUESTION	RESPONSE	CODE	JUMP
65.	During the last 6 months, has anyone from this home visited or worked in a hospital or clinic?	Less than once per week: 1<br>1 to 2 times per week: 2<br>3 or more times per week: 3<br>No: 4<br>Don't know: 96<br>No response: 999		___
66.	During the last 6 months, has anyone from this home worked outside the home with animals?	Less than once per week: 1<br>1 to 2 times per week: 2<br>3 or more times per week: 3<br>No: 4<br>Don't know: 96<br>No response: 999		If "4", "96" or "999" go to #68
67.	What animals have they worked with?	Name of animal(s): ______________________<br>Don't know/Don't remember: 96<br>No response: 999		___
68.	During the last 6 months, has anyone in your home slaughtered livestock or chickens, or processed animals or animal products such as meat, poultry, cheese, or milk for consumption or sale?	Less than once per week: 1<br>1 to 2 times per week: 2<br>3 or more times per week: 3<br>No: 4<br>Don't know: 96<br>No response: 999		___
69.	During the last 6 months, has anyone in your household worked with, treated or disposed of human or animal fecal waste not from your home?	Less than once per week: 1<br>1 to 2 times per week: 2<br>3 or more times per week: 3<br>No: 4<br>Don't know: 96<br>No response: 999		___

UNIVERSIDAD SAN FRANCISCO

Section G: Knowledge, attitudes and practices (KAP) about antibiotic use

Nº.	QUESTION	RESPONSE	CODE	JUMP
70.	When your child's throat hurts, do you give them antibiotics?	Always: 1<br>Sometimes: 2<br>Never: 3<br>Don't know: 96<br>No response: 999		—
71.	When your child catches a cold, will antibiotics help them improve more quickly?	Always: 1<br>Sometimes: 2<br>Never: 3<br>Don't know: 96<br>No response: 999		—
72.	When your child is sick with a bad cold, bad enough that they need to go to the doctor, do you expect the doctor to prescribe antibiotics?	Always: 1<br>Sometimes: 2<br>Never: 3<br>Don't know: 96<br>No response: 999		—
73.	Is it okay to use antibiotics when you feel sick, to help you get better?	Always: 1<br>Sometimes: 2<br>Never: 3<br>Don't know: 96<br>No response: 999		—
74.	If your child has bronchitis and you cannot take them to the doctor quickly, is it okay to go to a pharmacy to get antibiotics?	Always: 1<br>Sometimes: 2<br>Never: 3<br>Don't know: 96<br>No response: 999		—
75.	Do most of your friends think that they should give antibiotics to their children when they have a cold?	Always: 1<br>Sometimes: 2<br>Never: 3<br>Don't know: 96<br>No response: 999		—
76.	Do most of your friends think that they should give antibiotics to their children when they have diarrhea?	Always: 1<br>Sometimes: 2<br>Never: 3<br>Don't know: 96<br>No response: 999		—
77.	Do most of your friends think that they should give antibiotics to their children when they have skin rashes?	Always: 1<br>Sometimes: 2<br>Never: 3<br>Don't know: 96<br>No response: 999		—

78.	Do most of your friends get antibiotics for their children who are sick without a medical prescription?	Always: 1<br>Sometimes: 2<br>Never: 3<br>Don't know: 96<br>No response: 999		—
79.	Can antibiotics kill bacteria?	Yes: 1<br>No: 2<br>Don't know: 96<br>No response: 999		—
80.	Can antibiotics kill viruses?	Yes: 1<br>No: 2<br>Don't know: 96<br>No response: 999		—

Section H: Information about domestic pets, livestock and poultry

Nº.	QUESTION	RESPONSE	CODE	JUMP
81.	In the last 3 months, has your child had contact with domestic pets, livestock or poultry?	Less than once per week: 1<br>1 to 2 times per week: 2<br>3 or more times per week: 3<br>No: 4<br>Don't know: 96<br>No response: 999		—
82.	Do you currently have any animals on your property?	Yes: 1<br>No: 0<br>No response: 999		If "0" or "999" end the survey
83.	How many chickens do you have on your property?			—
84.	How many guinea pigs do you have on your property?			—
85.	How many pigs do you have on your property?			—
86.	How many cows/cattle do you have on your property?			—
87.	How many dogs do you have on your property?			—
88.	How many ducks/geese do you have on your property?			—
89.	How many sheep do you have on your property?			—
90.	How many rabbits do you have on your property?			—
91.	How many goats do you have on your property?			—
92.	How many cats do you have on your property?			—
93.	How many quail do you have on your property?			—
94.	Do you have other animals on your property? How many?			—
**Nº.**	**QUESTION**	**RESPONSE**	**CODE**	**JUMP**
95.	Do you allow pets or other animals in your home?	Less than once per week: 1<br>1 to 2 times per week: 2<br>3 or more times per week: 3<br>No: 4<br>Don't know: 96<br>No response: 999		—
96.	In the last 3 weeks, has any animal entered an area where your child spends time?	Less than once per week: 1<br>1 to 2 times per week: 2<br>3 or more times per week: 3<br>No: 4<br>Don't know: 96<br>No response: 999		—
97.	In the past 3 weeks, has your child played in areas where animals defecate?	Less than once per week: 1<br>1 to 2 times per week: 2<br>3 or more times per week: 3<br>No: 4		—

		Don't know: 96<br>No response: 999		
98.	How do you manage the feces from animals in your garden/patio/yard?	Leave it to decompose or break down: 1<br>Collect it and then place on land: 2<br>Use it on crops as a fertilizer: 3<br>Sell it: 4<br>Throw it away: 5<br>Does not apply: 6<br>Other: 5<br>Don't know: 96<br>No response: 999		—
99.	In the past 3 weeks, has your livestock and/or poultry consumed river or irrigation water?	Yes: 1<br>No: 2<br>Does not apply: 3<br>Don't know/ Don't remember: 96<br>No response: 999		—
100.	In the last 6 months, have you administered antibiotics to your animals, livestock or chickens?	Yes: 1<br>No: 0<br>Don't know/Don't remember: 96<br>No response: 999		If "0", "96" or "999" go to #106
101.	Where do you buy or obtain antibiotics for your animals?	Veterinarian: 1<br>Pet food store: 2<br>Pharmacy: 3<br>A family member or friend: 4<br>Other location: 5<br>Don't know/Don't remember: 96<br>No response: 999		—
102.	What type of instructions have you received about the use of antibiotics in animals?	Written instructions: 1<br>Oral instructions: 2<br>Have not received instructions: 3<br>Another professional gives them: 4<br>Don't know/Don't remember: 96<br>No response: 999		—
103.	To which animals have you given antibiotics?	Chickens: ☐<br>Guinea pigs: ☐<br>Swine: ☐<br>Cows/cattle: ☐<br>Dogs: ☐<br>Ducks/geese: ☐<br>Cheep: ☐<br>Rabbits: ☐<br>Goats: ☐<br>Cats: ☐<br>Quail: ☐<br>Other animals:☐		—
104.	How often do you give antibiotics to livestock, poultry or other animals?	As needed: 1<br>Routinely: 3		—

		Every day: 3<br>Don't know/Don't remember: 96<br>No response: 99		
105.	What are your reasons for using antibiotics in animals?<br>*Select all that apply*	Increases the growth of animals: ☐<br>Avoids the animals getting sick: ☐<br>Only give when animals are sick: ☐<br>It was recommended by a veterinarian: ☐<br>It was recommended by a pharmacist: ☐<br>Other: ☐<br>Don't know: ☐<br>No response: ☐		——
106.	Is it possible to see the antibiotic packaging?<br>*Take a picture with the Tablet*	Yes: 1<br>No: 2<br>Could not find: 3<br>No response: 99		——
107.	In the last 6 months, have you given any other medication or vitamins to your animals, livestock or chickens?	Yes: 1<br>No: 2<br>Don't know/Don't remember: 3<br>No response: 99		——
108.	Do you use commercial feed to feed any of your animals (for example, cattle, swine or poultry)?	Yes: 1<br>No: 2<br>Don't know: 3<br>No response: 99		If "0", "96" or "999" end the survey
109.	If so, what is the name?	Name: ______________________<br>Don't know: 96<br>No response: 999		——
110.	Is it possible to see the packaging?<br>*Take a picture with the Tablet*	Yes: 1<br>No: 2<br>Could not find: 3<br>No response: 99		——
