# Supplementary material for: Risk factors for third-generation cephalosporin-resistant and extended-spectrum β-lactamase-producing Escherichia coli carriage in domestic animals of semirural parishes east of Quito, Ecuador
Source: PLOS Glob Public Health. 2022 Mar 23;2(3):e0000206. doi: 10.1371/journal.pgph.0000206 (PMC10021719; doi:10.1371/journal.pgph.0000206)
Supplement: S1 Table — (PDF) [file pgph.0000206.s003.pdf]

**Risk Factor**	**Logistic regression model**	**Confounders**
Caregiver age	$\log(P_{AMR}/1\text{-}P_{AMR}) = \beta_0 + \beta_{age} + \beta_{education} + \beta_{SES}$	Caregiver education level, socioeconomic status (SES)
SES (household wealth)	$\log(P_{AMR}/1\text{-}P_{AMR}) = \beta_0 + \beta_{SES} + \beta_{education} + \beta_{\mathrm{age}}$	Caregiver education level, caregiver age
Household size	$\log(P_{AMR}/1\text{-}P_{AMR}) = \beta_0 + \beta_{household\ size} + \beta_{education} + \beta_{SES}$	Caregiver education level, SES
Highest level of caregiver education	$\log(P_{AMR}/1\text{-}P_{AMR}) = \beta_0 + \beta_{education} + \beta_{\mathrm{age}} + \beta_{SES}$	Caregiver age, SES
Proximity to nearest commercial food animal facility	$\log(P_{AMR}/1\text{-}P_{AMR}) = \beta_0 + \beta_{commercial\ facility\ proximity} + \beta_{education} + \beta_{SES}$	Caregiver education level, SES
Number of commercial food animal facilities within 5 km	$\log(P_{AMR}/1\text{-}P_{AMR}) = \beta_0 + \beta_{commercial\ facility\ density} + \beta_{education} + \beta_{SES}$	Caregiver education level, SES
Commercial poultry odors detected by respondent	$\log(P_{AMR}/1\text{-}P_{AMR}) = \beta_0 + \beta_{odors} + \beta_{education} + \beta_{SES} + \beta_{commercial\ facility\ proximity} + \beta_{commercial\ facility\ density}$	Caregiver education, SES, proximity to commercial food animal facility, density of commercial food animal facilities within 5 km
Number of animal species owned	$\log(P_{AMR}/1\text{-}P_{AMR}) = \beta_0 + \beta_{total\ species} + \beta_{education} + \beta_{SES} + \beta_{total\ animals}$	Caregiver education level, SES, total number of animals owned
Total number of animals	$\log(P_{AMR}/1\text{-}P_{AMR}) = \beta_0 + \beta_{total\ animals} + \beta_{education} + \beta_{SES} + \beta_{total\ species} + \beta_{food\ animals} + \beta_{vet\ access}$	Caregiver education level, SES, total number of animal species owned, total number of food animals owned, access to veterinary care
Total number of food animals	$\log(P_{AMR}/1\text{-}P_{AMR}) = \beta_0 + \beta_{food\ animals} + \beta_{education} + \beta_{SES} + \beta_{total\ species} + \beta_{total\ animals} + \beta_{water} + \beta_{vet\ access}$	Caregiver education level, SES, total number of animals owned, total number of animal species owned, animal water source (river/irrigation water), access to veterinary care
Livestock units owned	$\log(P_{AMR}/1\text{-}P_{AMR}) = \beta_0 + \beta_{livestock\ units} + \beta_{\mathrm{education}} + \beta_{SES} + \beta_{total\ species} + \beta_{total\ animals} + \beta_{water} + \beta_{vet\ access}$	Caregiver education level, SES (household wealth), total number of animals owned, total number of

		animal species owned, animal water source (irrigation/river water), access to veterinary care
Ownership of specific animal species (i.e. own dog, own chicken, etc.)	$\log(P_{AMR}/1-P_{AMR}) = \beta_0 + \beta_{type\ of\ species\ owned} + \beta_{education} + \beta_{SES} + \beta_{total\ animals} + \beta_{total\ species} + \beta_{water} + \beta_{vet\ access}$	Caregiver education level, SES, total number of animals owned, total number of species owned, water source (irrigation/river water), access to veterinary care
Antibiotic use in any animals in past 6 months	$\log(P_{AMR}/1-P_{AMR}) = \beta_0 + \beta_{\text{abx use}} + \beta_{education} + \beta_{SES} + \beta_{total\ animals} + \beta_{total\ species} + \beta_{vet\ access}$	Caregiver education level, SES (household wealth), total number of animals owned, total number of animal species owned, access to veterinary care
Antibiotic use specifically in dogs or food animals in past 6 months	$\log(P_{AMR}/1-P_{AMR}) = \beta_0 + \beta_{dog\ or\ food\ animal\ abx\ \text{use}} + \beta_{education} + \beta_{SES} + \beta_{total\ animals} + \beta_{total\ species} + \beta_{vet\ access}$	Caregiver education level, SES (household wealth), total number of animals owned, total number of animal species owned, access to veterinary care
Animals given other medications/vitamins in past 6 months	$\log(P_{AMR}/1-P_{AMR}) = \beta_0 + \beta_{other\ meds} + \beta_{education} + \beta_{SES} + \beta_{total\ animals} + \beta_{total\ species} + \beta_{vet\ access}$	Caregiver education level, SES, total number of animals owned, total number of animal species owned, access to veterinary care
Reason for using antibiotics	$\log(P_{AMR}/1-P_{AMR}) = \beta_0 + \beta_{abx\ reason} + \beta_{education} + \beta_{SES} + \beta_{total\ animals} + \beta_{total\ species} + \beta_{vet\ access}$	Caregiver education level, SES (household wealth), total number of animals owned, total number of animal species owned, access to veterinary care
Antibiotic source	$\log(P_{AMR}/1-P_{AMR}) = \beta_0 + \beta_{abx\ source} + \beta_{education} + \beta_{SES}$	Caregiver education level, SES
Veterinary access	$\log(P_{AMR}/1-P_{AMR}) = \beta_0 + \beta_{vet\ access} + \beta_{education} + \beta_{SES} + \beta_{total\ animals} + \beta_{total\ species}$	Caregiver education level, SES, total number of animals owned, total number of animal species owned
Animals consumed river or irrigation water in past 3 weeks	$\log(P_{AMR}/1-P_{AMR}) = \beta_0 + \beta_{water} + \beta_{education} + \beta_{SES} + \beta_{total\ animals}$	Caregiver education level, SES, total number of animals owned

Use of commercial feed	$\log(P_{AMR}/1\text{-}P_{AMR}) = \beta_0 + \beta_{commercial\ feed} + \beta_{education} + \beta_{SES} + \beta_{total\ animals} + \beta_{total\ species} + \beta_{food\ animals} + \beta_{vet\ access} + \beta_{commercial\ facility\ proximity}$	Caregiver education level, SES (household wealth), total number of animals owned, total number of animal species owned, total number of food animals owned, access to veterinary care, proximity to commercial food animal operation
Household member process animals or animal byproducts outside the home in past 6 months	$\log(P_{AMR}/1\text{-}P_{AMR}) = \beta_0 + \beta_{processes\ animals\ or\ byproducts} + \beta_{education} + \beta_{SES} + \beta_{commercial\ facility\ proximity}$	Caregiver education level, SES, proximity to commercial food animal facility
Household member works with animal or human feces outside the home in past 6 months	$\log(P_{AMR}/1\text{-}P_{AMR}) = \beta_0 + \beta_{works\ with\ feces} + \beta_{education} + \beta_{SES} + \beta_{commercial\ facility\ proximity}$	Caregiver education level, SES, proximity to commercial food animal facility
Household member took antibiotics in past 3 months	$\log(P_{AMR}/1\text{-}P_{AMR}) = \beta_0 + \beta_{education} + \beta_{total\ species} + \beta_{animal\ given\ antibiotics}$	Caregiver education level, total number of species owned, animal(s) given antibiotics in past 6 months
Animals allowed inside the home	$\log(P_{AMR}/1\text{-}P_{AMR}) = \beta_0 + \beta_{inside} + \beta_{education} + \beta_{SES} + \beta_{total\ animals} + \beta_{total\ species}$	Caregiver education level, SES, total number of animals owned, total number of species owned
Animals allowed near children	$\log(P_{AMR}/1\text{-}P_{AMR}) = \beta_0 + \beta_{near} + \beta_{education} + \beta_{SES} + \beta_{total\ animals} + \beta_{total\ species}$	Caregiver education level, SES, total number of animals owned, total number of species owned
Animal feces disposal practices	$\log(P_{AMR}/1\text{-}P_{AMR}) = \beta_0 + \beta_{feces} + \beta_{education} + \beta_{SES} + \beta_{total\ animals} + \beta_{total\ species} + \beta_{food\ animals}$	Caregiver education level, SES, total number of animals owned, total number of species owned, total number of food animals owned
Knowledge of whether or not antibiotics kill bacteria or viruses	$\log(P_{AMR}/1\text{-}P_{AMR}) = \beta_0 + \beta_{knowledge} + \beta_{education} + \beta_{SES}$	Caregiver education level, SES
