# Supplementary material for: Risk factors for third-generation cephalosporin-resistant and extended-spectrum β-lactamase-producing Escherichia coli carriage in domestic animals of semirural parishes east of Quito, Ecuador
Source: PLOS Glob Public Health. 2022 Mar 23;2(3):e0000206. doi: 10.1371/journal.pgph.0000206 (PMC10021719; doi:10.1371/journal.pgph.0000206)
Supplement: S2 Table — 1Fecal samples from “other” species, including llamas (2), cats (1), and hamsters (1), produced no CR E. coli isolates and so were not included in this table. 2Other poultry = geese, pigeon, and quail. 3Percentage of ceftriaxone-resistant (CR) E. coli isolates. 43GCR-MDR and 3GCR-XDR E. coli were determined from isolates resistant to ceftriaxone. (PDF) [file pgph.0000206.s004.pdf]

| | Dogs | | | Chickens | | | Guinea pigs | | | Pigs | | | Rabbits | | | Cows | | | Duck | | | Other poultry[2] | | | Sheep | | | Horse | | | Total | | |
|---|---|---|---|---|---|---|---|---|---|---|---|---|---|---|---|---|---|---|---|---|---|---|---|---|---|---|---|---|---|---|---|---|---|
| **Cycle** | 1 | 2 | 3 | 1 | 2 | 3 | 1 | 2 | 3 | 1 | 2 | 3 | 1 | 2 | 3 | 1 | 2 | 3 | 1 | 2 | 3 | 1 | 2 | 3 | 1 | 2 | 3 | 1 | 2 | 3 | 1 | 2 | 3 |
| **CR *E. coli* isolates** | 131 | 134 | 111 | 53 | 70 | 47 | 0 | 1 | 0 | 16 | 21 | 18 | 2 | 3 | 1 | 7 | 5 | 5 | 13 | 10 | 13 | 0 | 4 | 2 | 0 | 0 | 1 | 0 | 0 | 1 | 222 | 248 | 200 |
| **ESBL-producing *E. coli* (%)[3]** | 30 (23) | 25 (19) | 16 (14) | 8 (15) | 16 (23) | 5 (11) | 0 | 0 (0) | 0 | 3 (19) | 6 (29) | 3 (17) | 0 (0) | 2 (67) | 0 (0) | 0 (0) | 1 (20) | 0 (0) | 2 (15) | 1 (10) | 1 (8) | 0 | 3 (75) | 0 (0) | 0 | 0 | 0 (0) | 0 | 0 | 1 (100) | 43 (19) | 54 (22) | 26 (13) |
| **3GCR-MDR[4] *E. coli* (%)[3]** | 119 (91) | 126 (94) | 103 (93) | 47 (89) | 64 (91) | 40 (85) | 0 | 1 (100) | 0 | 13 (81) | 20 (95) | 17 (94) | 2 (100) | 2 (67) | 0 (0) | 5 (71) | 3 (60) | 5 (100) | 12 (92) | 9 (90) | 13 (100) | 0 | 4 (100) | 2 (100) | 0 | 0 | 1 (100) | 0 | 0 | 0 (0) | 198 (89) | 229 (92) | 181 (90) |
| **3GCR-XDR[4] *E. coli* (%)[3]** | 61 (47) | 66 (49) | 56 (50) | 20 (38) | 23 (33) | 22 (47) | 0 | 0 (0) | 0 | 6 (38) | 8 (38) | 7 (39) | 0 (0) | 0 (0) | 0 (0) | 2 (29) | 0 (0) | 1 (20) | 5 (38) | 1 (10) | 4 (31) | 0 | 2 (50) | 0 (0) | 0 | 0 | 0 (0) | 0 | 0 | 0 (0) | 94 (42) | 100 (40) | 90 (45) |
| **Cefazolin resistance (%)[3]** | 130 (99) | 134 (100) | 111 (100) | 53 (100) | 69 (99) | 47 (100) | 0 | 1 (100) | 0 | 16 (100) | 21 (100) | 18 (100) | 2 (100) | 3 (100) | 1 (100) | 7 (100) | 5 (100) | 5 (100) | 13 (100) | 10 (100) | 13 (100) | 0 | 4 (100) | 2 (100) | 0 | 0 | 1 (100) | 0 | 0 | 1 (100) | 221 (100) | 247 (100) | 200 (100) |
| **Ceftazidime resistance (%)[3]** | 45 (34) | 38 (28) | 22 (20) | 12 (23) | 26 (37) | 11 (23) | 0 | 0 (0) | 0 | 4 (25) | 11 (52) | 7 (39) | 0 (0) | 3 (100) | 0 (0) | 1 (14) | 1 (20) | 0 (0) | 2 (15) | 3 (30) | 1 (8) | 0 | 3 (75) | 0 (0) | 0 | 0 | 0 (0) | 0 | 0 | 1 (100) | 64 (29) | 85 (34) | 42 (21) |
| **Amoxicillin/clavulanic acid resistance (%)[3]** | 23 (18) | 16 (12) | 9 (8) | 9 (17) | 11 (16) | 7 (15) | 0 | 0 (0) | 0 | 1 (6) | 5 (24) | 4 (22) | 0 (0) | 1 (33) | 0 (0) | 1 (14) | 0 (0) | 0 (0) | 1 (8) | 2 (20) | 0 (0) | 0 | 1 (25) | 0 (0) | 0 | 0 | 0 (0) | 0 | 0 | 0 (0) | 35 (16) | 36 (15) | 20 (10) |
| **Cefotaxime resistance (%)[3]** | 124 (95) | 132 (99) | 107 (96) | 52 (98) | 69 (99) | 44 (94) | 0 | 1 (100) | 0 | 16 (100) | 19 (90) | 16 (89) | 2 (100) | 3 (100) | 1 (100) | 7 (100) | 5 (100) | 5 (100) | 13 (100) | 10 (100) | 13 (100) | 0 | 4 (100) | 2 (100) | 0 | 0 | 1 (100) | 0 | 0 | 1 (100) | 214 (96) | 243 (98) | 191 (96) |
| **Cefepime resistance (%)[3]** | 64 (49) | 79 (59) | 23 (21) | 30 (57) | 40 (57) | 15 (32) | 0 | 0 (0) | 0 | 9 (56) | 11 (52) | 6 (33) | 1 (50) | 3 (100) | 0 (0) | 3 (43) | 2 (40) | 1 (20) | 7 (54) | 5 (50) | 4 (31) | 0 | 2 (50) | 1 (50) | 0 | 0 | 0 (0) | 0 | 0 | 1 (100) | 114 (51) | 142 (57) | 51 (26) |
| **Ampicillin resistance (%)[3]** | 130 (99) | 132 (99) | 111 (100) | 53 (100) | 70 (100) | 47 (100) | 0 | 1 (100) | 0 | 16 (100) | 21 (100) | 18 (100) | 2 (100) | 3 (100) | 1 (100) | 7 (100) | 5 (100) | 5 (100) | 13 (100) | 10 (100) | 13 (100) | 0 | 4 (100) | 2 (100) | 0 | 0 | 1 (100) | 0 | 0 | 1 (100) | 221 (100) | 246 (99) | 200 (100) |
| **Gentamicin resistance (%)[3]** | 31 (24) | 39 (29) | 22 (20) | 10 (19) | 11 (16) | 10 (21) | 0 | 0 (0) | 0 | 4 (25) | 6 (29) | 3 (17) | 0 (0) | 1 (33) | 0 (0) | 1 (14) | 0 (0) | 1 (20) | 4 (31) | 1 (10) | 2 (15) | 0 | 1 (25) | 0 (0) | 0 | 0 | 0 (0) | 0 | 0 | 0 (0) | 50 (23) | 59 (24) | 38 (19) |
| **Imipenem resistance (%)[3]** | 0 (0) | 0 (0) | 0 (0) | 0 (0) | 1 (1) | 0 (0) | 0 | 0 (0) | 0 | 0 (0) | 0 (0) | 0 (0) | 0 (0) | 0 (0) | 0 (0) | 0 (0) | 0 (0) | 0 (0) | 0 (0) | 0 (0) | 0 (0) | 0 | 0 (0) | 0 (0) | 0 | 0 | 0 (0) | 0 | 0 | 0 (0) | 0 (0) | 1 (0) | 0 (0) |
| **Trimethoprim-sulfamethoxazole resistance (%)[3]** | 85 (65) | 98 (73) | 77 (69) | 31 (58) | 42 (60) | 32 (68) | 0 | 1 (100) | 0 | 9 (56) | 12 (57) | 14 (78) | 2 (100) | 1 (33) | 0 (0) | 3 (43) | 3 (60) | 3 (60) | 9 (69) | 6 (60) | 6 (46) | 0 | 3 (75) | 0 (0) | 0 | 0 | 1 (100) | 0 | 0 | 0 (0) | 139 (63) | 166 (67) | 133 (66) |
| **Tetracycline resistance (%)[3]** | 116 (89) | 111 (83) | 93 (84) | 40 (75) | 57 (81) | 38 (81) | 0 | 1 (100) | 0 | 11 (69) | 18 (86) | 16 (89) | 2 (100) | 1 (33) | 0 (0) | 5 (71) | 3 (60) | 3 (60) | 10 (77) | 9 (90) | 13 (100) | 0 | 4 (100) | 2 (100) | 0 | 0 | 1 (100) | 0 | 0 | 0 (0) | 184 (83) | 204 (82) | 166 (83) |
| **Ciprofloxacin resistance (%)[3]** | 67 (51) | 76 (56) | 64 (58) | 28 (53) | 28 (40) | 23 (49) | 0 | 0 (0) | 0 | 8 (50) | 12 (57) | 7 (39) | 0 (0) | 0 (0) | 0 (0) | 2 (29) | 0 (0) | 2 (40) | 5 (38) | 0 (0) | 3 (23) | 0 | 1 (25) | 0 (0) | 0 | 0 | 0 (0) | 0 | 0 | 0 (0) | 110 (50) | 116 (47) | 99 (50) |
