# Supplementary material for: Risk factors for third-generation cephalosporin-resistant and extended-spectrum β-lactamase-producing Escherichia coli carriage in domestic animals of semirural parishes east of Quito, Ecuador
Source: PLOS Glob Public Health. 2022 Mar 23;2(3):e0000206. doi: 10.1371/journal.pgph.0000206 (PMC10021719; doi:10.1371/journal.pgph.0000206)
Supplement: S3 Table — 13GCR-MDR and 3GCR-XDR E. coli were determined from isolates resistant to ceftriaxone. 2Odds ratio. 395% confidence interval. Bolded numbers indicate statistical significance (α = 0.05). 4Livestock units = (0.01) (number of chickens) + (0.30) (number of pigs) + (0.80) (number of cattle) + (0.10) (number of sheep) + (0.10) (number of goats) + (0.02) (number of rabbits) + (0.01) (number of guinea pigs) + (0.03) (number of ducks) + (0.03) (number of quail). (PDF) [file pgph.0000206.s005.pdf]

Risk Factor	CR *E. coli*		ESBL-producing *E.coli*		3GCR-MDR *E. coli* [1]		3GCR-XDR *E. coli* [1]	
	**Unadjusted OR[2]**	**95% CI[3]**	**Unadjusted OR[2]**	**95% CI[3]**	**Unadjusted OR[2]**	**95% CI[3]**	**Unadjusted OR[2]**	**95% CI[3]**
Caregiver age								
<30 years old (n=305)	Reference							
≥30 years old (n=250)	0.79	0.55-1.13	1.00	0.61-1.65	0.79	0.56-1.12	0.95	0.67-1.36
Household wealth								
Low (n=166)	Reference							
Medium/ High (n=389)	1.00	0.68-1.48	1.83	0.99-3.37	0.99	0.68-1.45	0.92	0.62-1.34
Household Size								
1-5 members (n=433)	Reference							
>5 members (n=122)	1.19	0.77-1.85	1.12	0.62-2.01	1.12	0.73-1.70	1.04	0.68-1.59
Highest level of caregiver education								
Elementary (n=164)	Reference							
High School/ University (n=391)	1.25	0.85-1.83	1.27	0.72-2.25	1.17	0.80-1.70	1.18	0.79-1.74
Proximity to nearest commercial food animal facility								
>2 km (n=117)	Reference							
1-2 km (n=211)	1.35	0.84-2.16	1.22	0.61-2.46	1.43	0.90-2.27	1.32	0.81-2.15
<1 km (n=227)	1.41	0.88-2.25	1.23	0.61-2.45	1.29	0.82-2.03	1.20	0.74-1.96
Commercial food animal facilities within 5 km								
0-5 (n=135)	Reference							
>5 (n=419)	**1.67**	**1.12-2.50**	1.51	0.80-2.85	**1.70**	**1.14-2.52**	1.29	0.84-1.97
Commercial poultry odors detected by respondent								
No/don't know (n=389)	Reference							
Yes (n=167)	**1.81**	**1.19-2.74**	0.97	0.56-1.66	**1.82**	**1.22-2.70**	1.12	0.76-1.64
Number of species at household								
1-3 (n=422)	Reference							
>3 (n=130)	0.97	0.64-1.48	1.32	0.76-2.31	0.93	0.62-1.40	1.09	0.72-1.65
Number of animals at household								
1-5 (n=317)	Reference							
6-20 (n=134)	0.99	0.64-1.53	1.01	0.55-1.85	0.82	0.54-1.24	0.94	0.62-1.45
>20 (n=101)	0.94	0.58-1.52	1.12	0.59-2.14	0.93	0.58-1.48	1.01	0.63-1.62
Number of food animals at household								
None (n=291)	Reference							
1-10 (n=126)	0.95	0.61-1.49	1.48	0.81-2.69	0.87	0.57-1.34	0.86	0.55-1.36
>10 (n=136)	1.10	0.70-1.70	1.20	0.65-2.21	1.03	0.67-1.57	1.10	0.71-1.68
Livestock units at household[4]								
None (n=291)	Reference							
0-1 (n=202)	1.07	0.73-1.58	1.53	0.91-2.57	0.95	0.66-1.38	0.95	0.65-1.40
>1 (n=60)	0.89	0.49-1.59	0.71	0.27-1.90	0.92	0.52-1.63	1.08	0.60-1.93
Own cat(s)								
No (n=398)	Reference							
Yes (n=156)	1.12	0.75-1.67	1.09	0.63-1.87	1.01	0.69-1.48	0.98	0.66-1.45
Own chicken(s)								
No (n=328)	Reference							
Yes (n=228)	0.86	0.60-1.23	1.06	0.64-1.75	0.82	0.58-1.16	1.03	0.72-1.48
Own guinea pig(s)								
No (n=439)	Reference							

Yes (n=115)	1.14	0.73-1.79	0.94	0.50-1.74	1.16	0.75-1.79	0.90	0.58-1.41
Own pig(s)								
No (n=478)	Reference							
Yes (n=76)	0.87	0.52-1.46	0.89	0.42-1.88	0.92	0.55-1.51	0.64	0.37-1.11
Own rabbit(s)								
No (n=491)	Reference							
Yes (n=64)	1.10	0.62-1.96	1.31	0.64-2.68	1.08	0.63-1.88	0.91	0.52-1.60
Own duck(s)								
No (n=499)	Reference							
Yes (n=56)	1.17	0.63-2.15	0.63	0.24-1.65	1.03	0.58-1.83	1.61	0.91-2.84
Own cow(s)								
No (n=510)	Reference							
Yes (n=45)	0.93	0.49-1.78	0.64	0.22-1.86	1.07	0.57-2.03	1.40	0.75-2.60
