# Supplementary material for: Risk factors for third-generation cephalosporin-resistant and extended-spectrum β-lactamase-producing Escherichia coli carriage in domestic animals of semirural parishes east of Quito, Ecuador
Source: PLOS Glob Public Health. 2022 Mar 23;2(3):e0000206. doi: 10.1371/journal.pgph.0000206 (PMC10021719; doi:10.1371/journal.pgph.0000206)
Supplement: S5 Table — 13GCR-MDR and 3GCR-XDR E. coli were determined from isolates resistant to ceftriaxone. 2Odds ratio. 395% confidence interval. Bolded numbers indicate statistical significance (α = 0.05). 4Livestock units = (0.01) (number of chickens) + (0.30) (number of pigs) + (0.80) (number of cattle) + (0.10) (number of sheep) + (0.10) (number of goats) + (0.02) (number of rabbits) + (0.01) (number of guinea pigs) + (0.03) (number of ducks) + (0.03) (number of quail). (PDF) [file pgph.0000206.s007.pdf]

Risk Factor	3GCR-MDR *E. coli* [1]		3GCR-XDR *E. coli* [1]	
	**Adjusted OR[2]**	**95% CI[3]**	**Adjusted OR[2]**	**95% CI[3]**
Caregiver age				
<30 years old (n=305)	Reference			
≥30 years old (n=250)	0.81	0.56-1.19	1.02	0.70-1.49
Household wealth				
Low (n=166)	Reference			
Medium/High (n=389)	1.02	0.69-1.49	0.91	0.62-1.35
Household Size				
1-5 members (n=433)	Reference			
>5 members (n=122)	1.14	0.74-1.74	1.05	0.69-1.62
Highest level of caregiver education				
Elementary (n=164)	Reference			
High School/ University (n=391)	1.08	0.71-1.63	1.18	0.78-1.80
Proximity to nearest commercial food animal facility				
>2 km (n=117)	Reference			
1-2 km (n=211)	1.45	0.91-2.31	1.34	0.82-2.18
<1 km (n=227)	1.30	0.83-2.06	1.23	0.76-1.99
Commercial food animal facilities within 5 km				
0-5 (n=135)	Reference			
>5 (n=419)	**1.73**	**1.17-2.58**	1.32	0.86-2.01
Commercial poultry odors detected by respondent				
No/don't know (n=388)	Reference			
Yes (n=166)	**1.89**	**1.26-2.83**	1.17	0.79-1.73
Number of species at household				
1-3 (n=422)	Reference			
>3 (n=130)	1.01	0.55-1.86	1.22	0.65-2.31
Number of animals at household				
1-5 (n=312)	Reference			
6-20 (n=131)	0.63	0.32-1.26	0.83	0.40-1.72
>20 (n=95)	0.51	0.17-1.53	0.68	0.23-2.08
Number of food animals at household				
None (n=285)	Reference			
1-10 (n=124)	1.18	0.62-2.26	0.92	0.48-1.78
>10 (n=129)	2.00	0.69-5.80	1.37	0.49-3.85
Livestock units at household[4]				
None (n=285)	Reference			
> 0 and ≤ 1 (n=197)	1.22	0.63-2.34	0.94	0.49-1.80
>1 (n=56)	1.22	0.44-3.44	1.12	0.41-3.02
Own cat(s)				
No (n=389)	Reference			
Yes (n=149)	1.00	0.65-1.54	0.92	0.59-1.44
Own chicken(s)				
No (n=319)	Reference			
Yes (n=219)	0.82	0.43-1.56	1.18	0.63-2.20
Own guinea pig(s)				
No (n=428)	Reference			
Yes (n=110)	1.49	0.76-2.93	0.60	0.31-1.18
Own pig(s)				
No (n=465)	Reference			
Yes (n=73)	1.00	0.55-1.81	**0.51**	**0.26-0.98**
Own rabbit(s)				

No (n=479)	Reference			
Yes (n=59)	1.02	0.54-1.92	0.72	0.37-1.39
Own duck(s)				
No (n=484)	Reference			
Yes (n=54)	1.10	0.55-2.17	**2.09**	**1.02-4.27**
Own cow(s)				
No (n=497)	Reference			
Yes (n=41)	1.16	0.54-2.48	1.70	0.78-3.69
