# Supplementary material for: Risk factors for third-generation cephalosporin-resistant and extended-spectrum β-lactamase-producing Escherichia coli carriage in domestic animals of semirural parishes east of Quito, Ecuador
Source: PLOS Glob Public Health. 2022 Mar 23;2(3):e0000206. doi: 10.1371/journal.pgph.0000206 (PMC10021719; doi:10.1371/journal.pgph.0000206)
Supplement: S6 Table — 13GCR-MDR and 3GCR-XDR E. coli were determined from isolates resistant to ceftriaxone. 2Odds ratio. 395% confidence interval. Bolded numbers indicate statistical significance (α = 0.05). 4Questions regarding motivation for antibiotic use and antibiotic source were only answered by those caregivers that reported using antibiotics for their animal(s). 5Household member use of antibiotics was determined based on caregiver response to whether or not their child in the study had taken antibiotics in the past 3 months and whether or not a household member had taken antibiotics in the past 3 months, the latter of which was only asked to those who reported having a household member with an illness or infection in the past 3 months. (PDF) [file pgph.0000206.s008.pdf]

Risk Factor	3GCR-MDR *E. coli* [1]		3GCR-XDR *E. coli* [1]	
	**Adjusted OR[2]**	**95% CI[3]**	**Adjusted OR[2]**	**95% CI[3]**
Antibiotics given to any animals in past 6 months				
No (n=467)	Reference			
Yes (n=71)	**4.35**	**1.23-15.39**	1.83	0.78-4.30
Antibiotics given to dogs in past 6 months				
No (n=510)	Reference			
Yes (n=28)	**2.85**	**1.10-7.43**	1.42	0.54-3.74
Other medications/vitamins given in past 6 months				
No (n=440)	Reference			
Yes (n=96)	1.06	0.66-1.69	0.91	0.56-1.47
Use antibiotics for growth promotion[4]				
No (n=44)	Reference			
Yes (n=21)	0.75	0.24-2.35	1.08	0.30-3.88
Use antibiotics for animal illness prevention[4]				
No (n=49)	Reference			
Yes (n=17)	0.87	0.19-3.95	1.18	0.34-4.09
Use antibiotics for animal illness treatment[4]				
No (n=55)	Reference			
Yes (n=9)	2.41	0.40-14.42	0.36	0.07-1.84
Use antibiotics based on veterinary/ pharmacy recommendation[4]				
No (n=55)	Reference			
Yes (n=11)	0.22	0.04-1.19	0.82	0.17-4.00
Antibiotic Source[4]				
Veterinarian (n=47)			Reference	
Pet food store (n=17)	3.32	0.82-13.47	2.05	0.61-6.91
Veterinary access				
No (n=490)	Reference			
Yes (n=48)	0.79	0.43-1.45	0.80	0.41-1.54
Animals consumed river or irrigation water in past 3 weeks				
No (n=508)	Reference			
Yes (n=44)	1.06	0.55-2.06	0.92	0.46-1.82
Animals fed commercial feed				
No/Don't know (n=348)	Reference			
Yes (n=184)	**0.67**	**0.45-0.996**	0.93	0.62-1.40
Household member slaughtered livestock/poultry, worked with animals, or worked in animal or animal by-product processing in past 6 months				
No/Don't know (n=413)	Reference			
Yes (n=142)	1.48	0.98-2.25	1.19	0.80-1.77
Household member worked with animal or human feces outside the home in past 6 months				
No/Don't know (n=494)	Reference			
Yes (n=61)	0.92	0.52-1.60	1.08	0.62-1.88
Household member took antibiotics in past 3 months[5]				
No (n=33)	Reference			
Yes (n=138)	1.00	0.44-2.28	1.11	0.49-2.49
Animals allowed inside the home				
No/Don't know (n=286)	Reference			
Yes (n=263)	1.15	0.81-1.64	1.13	0.79-1.63
Animals allowed near children				
No/Don't know (n=216)	Reference			
Yes (n=334)	1.29	0.91-1.85	1.02	0.71-1.48
Animal feces management				
Place in trash (n=264)	Reference			

Leave in yard (n=99)	1.01	0.62-1.64	0.69	0.41-1.16
Store and place on land/ Use as fertilizer (n=133)	1.16	0.69-1.93	0.74	0.44-1.24
Can antibiotics kill bacteria?				
"Yes"/Correct (n=210)	Reference			
"No"/Incorrect (n=113)	0.85	0.53-1.36	0.76	0.47-1.25
Don't know (n=232)	1.08	0.73-1.60	0.82	0.55-1.23
Can antibiotics kill viruses?				
"No"/Correct (n=116)	Reference			
"Yes"/Incorrect (n=207)	1.10	0.69-1.77	1.44	0.87-2.37
Don't know (n=231)	1.20	0.75-1.91	1.23	0.74-2.03
