# Supplementary material for: Risk factors for third-generation cephalosporin-resistant and extended-spectrum β-lactamase-producing Escherichia coli carriage in domestic animals of semirural parishes east of Quito, Ecuador
Source: PLOS Glob Public Health. 2022 Mar 23;2(3):e0000206. doi: 10.1371/journal.pgph.0000206 (PMC10021719; doi:10.1371/journal.pgph.0000206)
Supplement: S7 Table — 13GCR-MDR and 3GCR-XDR E. coli were determined from isolates resistant to ceftriaxone. 2Odds ratio. 395% confidence interval. Bolded numbers indicate statistical significance (α = 0.05). 4Livestock units = (0.01) (number of chickens) + (0.30) (number of pigs) + (0.80) (number of cattle) + (0.10) (number of sheep) + (0.10) (number of goats) + (0.02) (number of rabbits) + (0.01) (number of guinea pigs) + (0.03) (number of ducks) + (0.03) (number of quail). (PDF) [file pgph.0000206.s009.pdf]

Risk Factor	CR *E. coli*		ESBL-producing *E.coli*		3GCR-MDR *E. coli*[1]		3GCR-XDR *E. coli*[1]	
	Unadjusted OR[2]	95% CI[3]	Unadjusted OR[2]	95% CI[3]	Unadjusted OR[2]	95% CI[3]	Unadjusted OR[2]	95% CI[3]
Caregiver age								
<30 years old (n=354)	Reference							
≥30 years old (n=283)	1.19	0.89-1.58	0.70	0.39-1.25	1.09	0.82-1.44	0.86	0.57-1.29
Household wealth								
Low (n=222)	Reference							
Medium/High (n=415)	0.86	0.63-1.17	0.77	0.43-1.40	0.88	0.65-1.19	1.00	0.66-1.52
Household Size								
1-5 members (n=480)	Reference							
>5 members (n=157)	1.06	0.76-1.48	**2.24**	**1.26-4.01**	1.16	0.83-1.62	0.97	0.59-1.59
Highest level of caregiver education								
Elementary (n=211)	Reference							
High School/ University (n=426)	1.11	0.81-1.51	0.71	0.40-1.27	1.25	0.93-1.67	**1.96**	**1.17-3.27**
Proximity to nearest commercial food animal facility								
>2 km (n=159)	Reference							
1-2 km (n=197)	0.80	0.54-1.19	1.93	0.79-4.72	0.77	0.52-1.12	0.65	0.38-1.10
<1 km (n=281)	0.92	0.62-1.37	**2.53**	**1.11-5.76**	0.83	0.58-1.20	0.88	0.56-1.38
Commercial food animal facilities within 5 km								
0-5 (n=134)	Reference							
>5 (n=503)	1.07	0.75-1.51	1.30	0.64-2.63	1.03	0.74-1.44	1.02	0.63-1.67
Commercial poultry odors detected by respondent								
No/don't know (n=395)	Reference							
Yes (n=242)	0.89	0.66-1.21	0.51	0.26-1.01	0.98	0.72-1.31	0.94	0.62-1.43
Number of species at household								
1-3 (n=189)	Reference							
>3 (n=444)	**0.70**	**0.50-0.97**	0.87	0.47-1.58	0.79	0.57-1.10	0.78	0.51-1.19
Number of animals at household								
1-5 (n=53)	Reference							
6-20 (n=222)	0.86	0.48-1.54	0.73	0.28-1.92	0.74	0.41-1.31	0.61	0.30-1.25
>20 (n=358)	0.81	0.46-1.41	0.64	0.25-1.61	0.71	0.41-1.25	0.62	0.32-1.22
Number of food animals at household								
≤ 10 (n=188)	Reference							
11-20 (n=106)	0.83	0.54-1.27	1.25	0.55-2.80	0.84	0.55-1.29	0.74	0.39-1.40
>20 (n=339)	0.88	0.64-1.23	0.86	0.45-1.64	0.90	0.65-1.24	0.83	0.53-1.31
Livestock units at household[4]								
0-1 (n=363)	Reference							
>1 (n=270)	1.10	0.83-1.47	**0.51**	**0.29-0.92**	1.11	0.84-1.47	0.83	0.55-1.24
Own dog(s)								
No (n=51)	Reference							
Yes (n=586)	0.72	0.41-1.25	0.52	0.22-1.23	0.77	0.46-1.30	1.05	0.49-2.23
Own cat(s)								
No (n=341)	Reference							
Yes (n=296)	1.06	0.79-1.41	1.27	0.72-2.24	1.17	0.89-1.55	1.09	0.73-1.63
Own chicken(s)								
No (n=70)	Reference							
Yes (n=567)	**2.72**	**1.58-4.68**	1.18	0.45-3.05	**2.59**	**1.46-4.63**	1.81	0.81-4.03

Own guinea pig(s)								
No (n=248)	Reference							
Yes (n=386)	**0.56**	**0.41-0.78**	0.73	0.41-1.28	**0.62**	**0.46-0.85**	0.76	0.51-1.14
Own pig(s)								
No (n=344)	Reference							
Yes (n=292)	1.31	0.99-1.74	1.10	0.62-1.94	**1.34**	**1.01-1.77**	1.20	0.81-1.79
Own rabbit(s)								
No (n=401)	Reference							
Yes (n=236)	**0.63**	**0.46-0.85**	0.60	0.31-1.16	**0.72**	**0.53-0.98**	0.76	0.50-1.15
Own duck(s)								
No (n=428)	Reference							
Yes (n=209)	**1.41**	**1.04-1.90**	0.90	0.49-1.67	**1.45**	**1.08-1.93**	1.24	0.83-1.85
Own cow(s)								
No (n=428)	Reference							
Yes (n=209)	1.19	0.88-1.61	0.74	0.41-1.32	1.23	0.92-1.63	0.92	0.61-1.40
