# Supplementary material for: Risk factors for third-generation cephalosporin-resistant and extended-spectrum β-lactamase-producing Escherichia coli carriage in domestic animals of semirural parishes east of Quito, Ecuador
Source: PLOS Glob Public Health. 2022 Mar 23;2(3):e0000206. doi: 10.1371/journal.pgph.0000206 (PMC10021719; doi:10.1371/journal.pgph.0000206)
Supplement: S9 Table — 13GCR-MDR and 3GCR-XDR E. coli were determined from isolates resistant to ceftriaxone. 2Odds ratio. 395% confidence interval. Bolded numbers indicate statistical significance (α = 0.05). 4Livestock units = (0.01) (number of chickens) + (0.30) (number of pigs) + (0.80) (number of cattle) + (0.10) (number of sheep) + (0.10) (number of goats) + (0.02) (number of rabbits) + (0.01) (number of guinea pigs) + (0.03) (number of ducks) + (0.03) (number of quail). (PDF) [file pgph.0000206.s011.pdf]

Risk Factor	3GCR-MDR *E. coli*[1]		3GCR-XDR *E. coli*[1]	
	**Adjusted OR[2]**	**95% CI[3]**	**Adjusted OR[2]**	**95% CI[3]**
Caregiver age				
<30 years old (n=354)	Reference			
≥30 years old (n=283)	1.27	0.91-1.77	1.13	0.74-1.72
Household wealth				
Low (n=222)	Reference			
Medium/High (n=415)	0.87	0.64-1.17	1.01	0.67-1.53
Household Size				
1-5 members (n=480)	Reference			
>5 members (n=157)	1.16	0.83-1.63	0.99	0.62-1.58
Highest level of caregiver education				
Elementary (n=211)			Reference	
High School/ University (n=426)	1.39	0.99-1.96	**2.07**	**1.22-3.53**
Proximity to nearest commercial food animal facility				
>2 km (n=159)	Reference			
1-2 km (n=197)	0.81	0.55-1.19	0.74	0.43-1.26
<1 km (n=281)	0.89	0.61-1.28	1.00	0.65-1.55
Commercial food animal facilities within 5 km				
0-5 (n=134)	Reference			
>5 (n=503)	1.06	0.76-1.47	1.07	0.66-1.72
Commercial poultry odors detected by respondent				
No/don't know (n=395)	Reference			
Yes (n=242)	0.97	0.72-1.29	0.92	0.61-1.37
Number of species at household				
1-3 (n=189)	Reference			
>3 (n=444)	0.82	0.53-1.24	0.83	0.47-1.46
Number of animals at household				
1-5 (n=53)	Reference			
6-20 (n=214)	0.82	0.43-1.57	0.70	0.32-1.52
>20 (n=342)	0.58	0.21-1.61	0.78	0.20-3.12
Number of food animals at household				
≤ 10 (n=183)	Reference			
11-20 (n=103)	1.03	0.61-1.73	1.00	0.44-2.27
>20 (n=323)	1.47	0.62-3.53	0.84	0.23-3.09
Livestock units at household[4]				
≤ 1 (n=355)	Reference			
>1 (n=254)	1.23	0.85-1.79	0.69	0.42-1.15
Own dog(s)				
No (n=48)	Reference			
Yes (n=561)	0.89	0.52-1.55	1.06	0.50-2.26
Own cat(s)				
No (n=331)	Reference			
Yes (n=278)	1.31	0.98-1.76	1.24	0.80-1.93
Own chicken(s)				
No (n=63)	Reference			
Yes (n=546)	**5.00**	**2.33-10.74**	**2.95**	**1.28-6.80**
Own guinea pig(s)				
No (n=236)	Reference			
Yes (n=373)	**0.59**	**0.40-0.88**	0.87	0.53-1.44
Own pig(s)				
No (n=333)	Reference			
Yes (n=276)	**1.52**	**1.11-2.08**	1.19	0.75-1.88

Own rabbit(s)				
No (n=387)	Reference			
Yes (n=222)	**0.69**	**0.50-0.96**	0.73	0.47-1.12
Own duck(s)				
No (n=409)	Reference			
Yes (n=200)	**1.72**	**1.25-2.37**	1.23	0.79-1.94
Own cow(s)				
No (n=416)	Reference			
Yes (n=193)	1.42	1.00-2.02	0.90	0.56-1.45
