# Supplementary material for: Risk factors for third-generation cephalosporin-resistant and extended-spectrum β-lactamase-producing Escherichia coli carriage in domestic animals of semirural parishes east of Quito, Ecuador
Source: PLOS Glob Public Health. 2022 Mar 23;2(3):e0000206. doi: 10.1371/journal.pgph.0000206 (PMC10021719; doi:10.1371/journal.pgph.0000206)
Supplement: Attachment — Submitted filename: Mitmanetal_3GCRandESBLinAnimals_ResponsestoReviewers.docx [file pgph.0000206.s013.docx]

**Reviewer Comments and Responses**

Thank you for your time and commitment to reviewing our manuscript. We appreciate your thoughtful and thorough insight. Below, please find our responses to each question or concern raised in the first-round review process. Reviewer/journal comments can be found in italics and our responses in regular font.

*1. Please include additional information regarding the survey or questionnaire used in the study and ensure that you have provided sufficient details that others could replicate the analyses. For instance, if you developed a questionnaire as part of this study and it is not under a copyright more restrictive than CC-BY, please include a copy, in both the original language and English, as Supporting Information.*

We have now added the survey in English and Spanish as part of the supplemental information (S1 and S2 Survey).

*2. If you have no competing interests to declare, please state "The authors have declared that no competing interests exist".*

 This statement has been updated under the “Conflict of Interest” heading.

*3. Thank you for providing the following URL in your Data Availability Statement:* [*https://doi.org/10.6078/D1ZM6F*](https://doi.org/10.6078/D1ZM6F)

*Unfortunately, we are unable to access the URL as is. Please provide an updated, functional URL or DOI for the data underlying your study. We will update your Data Availability statement on your behalf to reflect the information you provide.*

Our apologies that this link did not work. The reviewer URL for this dataset can be found here: <https://datadryad.org/stash/share/69p_cRy7Xs81-3Nwkcdsukx_xldRgrY7c6dhcm0eEeI>

Following publication of this manuscript, the data will be available here:

Mitman, Siena (2021), MITMAN_PRISA_AMRinAnimalsEcuador, Dryad, Dataset, <https://doi.org/10.6078/D1ZM6F>

*4. Please amend your detailed Financial Disclosure statement. This is published with the article, therefore should be completed in full sentences and contain the exact wording you wish to be published.*

*i). State the initials, alongside each funding source, of each author to receive each grant.*

*ii). State what role the funders took in the study. If the funders had no role in your study, please state: “The funders had no role in study design, data collection and analysis, decision to publish, or preparation of the manuscript.”*

We have now provided this information for each of the authors and included the statement, “The funders had no role in study design, data collection and analysis, decision to publish, or preparation of the manuscript.”

*Please review your reference list to ensure that it is complete and correct. If you have cited papers that have been retracted, please include the rationale for doing so in the manuscript text, or remove these references and replace them with relevant current references. Any changes to the reference list should be mentioned in the rebuttal letter that accompanies your revised manuscript. If you need to cite a retracted article, indicate the article’s retracted status in the References list and also include a citation and full reference for the retraction notice.*

The citations for this manuscript have been reviewed. All in-text citations appear in the reference list and vice versa. The following changes were made to the reference list:

The following Erratum was added to the citation below:

Li J, Bi Z, Ma S, Chen B, Cai C, He J, et al. Inter-host Transmission of Carbapenemase Producing Escherichia coli among Humans and Backyard Animals. Environ Health Perspect. 2019 Oct;127(10):107009.

Li J, Bi Z, Ma S, Chen B, Cai C, He J, et al. Erratum: “Inter-Host Transmission of Carbapenemase-Producing Escherichia coli among Humans and Backyard Animals”. Environ Health Perspect. 2020 Jan;128(1):19001.

The following citation was removed as it is not yet in press:

Loayza-Villa F, Torres A, Zhang L, Trueba G. Withdraw of prophylactic antimicrobials does not change the pigs’ resistome. BioRxiv [Preprint]. 2019 bioRxiv 716597 [posted 2019 July 26]: [17 p.]: Available from: https://www.biorxiv.org/content/10.1101/716597v1.full doi: 10.1101/716597.

The following citation has been updated given its progression from in press to published:

Kurowski K, Marusinec R, Amato H, Saraiva-Garcia C, Loayza F, Salinas L, et al. Social and environmental determinants of community-acquired antimicrobial resistant E. coli in children living in semirural communities of Quito, Ecuador. Am J Trop Med Hyg. 2021;105(3):600–10.

The following citation was removed as it was a thesis and did not represent a peer-reviewed publication:

Suárez BDS. Determinantes para el uso de Antibióticos Según las Actitudes y Percepciones de Médicos Veterinarios en la Producción Avícola de Ecuador. Título de Médico Veterinario y Zootecnista. Thesis. Universidad Central Del Ecuador, Quito, Ecuador. 2019. Available from: http://www.dspace.uce.edu.ec/bitstream/25000/18750/1/T-UCE-0014-MVE-053.pdf

**Comments to the Author**

*1. Does this manuscript meet PLOS Global Public Health’s* [*publication criteria*](https://journals.plos.org/globalpublichealth/s/criteria-for-publication)*? Is the manuscript technically sound, and do the data support the conclusions? The manuscript must describe methodologically and ethically rigorous research with conclusions that are appropriately drawn based on the data presented.*

Reviewer #1: Yes

Reviewer #2: Yes

*2. Has the statistical analysis been performed appropriately and rigorously?*

Reviewer #1: Yes

Reviewer #2: Yes

*3. Have the authors made all data underlying the findings in their manuscript fully available (please refer to the Data Availability Statement at the start of the manuscript PDF file)?

The* [*PLOS Data policy*](https://journals.plos.org/globalpublichealth/s/data-availability) *requires authors to make all data underlying the findings described in their manuscript fully available without restriction, with rare exception. The data should be provided as part of the manuscript or its supporting information, or deposited to a public repository. For example, in addition to summary statistics, the data points behind means, medians and variance measures should be available. If there are restrictions on publicly sharing data—e.g. participant privacy or use of data from a third party—those must be specified.*

Reviewer #1: Yes

Reviewer #2: Yes

*4. Is the manuscript presented in an intelligible fashion and written in standard English?

PLOS Global Public Health does not copyedit accepted manuscripts, so the language in submitted articles must be clear, correct, and unambiguous. Any typographical or grammatical errors should be corrected at revision, so please note any specific errors here.*

Reviewer #1: Yes

Reviewer #2: Yes

**Reviewer Comments to the Author**

Reviewer #1:

*L106: “small scale food animals” is confusing. Try “food animals from small-scale production facilities/small farms”*

This has been updated.

*L129-130: please provide more information on the study site. How were these parishes selected? What is the definition of a “parish”? What percentage of the parishes around Quito do these 7 represent? Etc.*

There are 32 urban parishes and 33 rural or suburban parishes that make up Quito, Ecuador. We selected 7 parishes because we believe these parishes are representative of communities that both live near large major metropolitan areas and maintain many rural practices, including small-scale food animal production. We also selected these parishes based on their proximity to the laboratory at La Universidad San Francisco de Quito (samples could be analyzed on the same day they were collected). This information has been added to the Materials and Methods section of this manuscript.

*L138: Please provide details on or a citation for the validation process.*

To validate this survey, we first established face validity by having field staff who live in the community evaluate the questions and determine whether they successfully captured the intended purpose. We then pilot tested the survey with community residents to address issues of comprehension and made revisions. This information has been added to the manuscript under the “Knowledge, attitudes and practices (KAP) survey” subheading.

*L148: please explain whether samples were collected during each cycle, or whether one sample/pooled samples was collected per household across all three time units. If samples were collected from each household at more than one time point, how were the outcomes treated? Ever positive for the ESBL E. coli? What if a sample was positive on one cycle then negative at another cycle?*

This has been clarified in the Materials and Methods section of the manuscript.

*L167: check grammar of this sentence.*

The grammar of this sentence has been revised.

*L207-212: I am not familiar with this unit. Please provide a brief explanation for why it is used, and why it would not be more useful to simply include a tally of the number of animals on the farm.*

Livestock units is an approach recommended by several agricultural organizations, such as the UN FAO. Using livestock units helps to account for the size of animals (and the fecal waste the animals produce). The unit is useful for understanding the scale of food-animal production and allows one to compare households with different types of animals (1 cow versus 100 guinea pigs, for example). This explanation has now been included in the manuscript.

*L216: I don’t know the size of these parishes and how close the households were to each other, but I can imagine the outcomes would be affected by clustering within a parish or within a certain geographic location of that parish. Did you assess /account for any clustering in your models? If not, please consider including this as a limitation. Was adjustment for multiple comparisons performed?*

We were not able to adjust for clustering and did not adjust for multiple comparisons, as this was an exploratory analysis to identify potential risk factors for AMR in livestock. We have added these important points to our limitations section.

*Table 1: Please consider indicating in the table whether the distribution of these variables were significantly different across the cycles.*

Table 1 has been updated to include p-values, calculated using Chi-square goodness of fit tests, to indicate significant differences for each variable across study cycles.

*L280: how was “Access to veterinary care” defined?*

This has now been defined in this sentence.

*L338: check your spelling of “cefazolin”*

This spelling has been corrected throughout the paper.

*Table 5: Do you have information on whether any human household members took antibiotics in the recent past?*

We have now added this information into the manuscript and tested it as a potential risk factor.

*Table 6: is there a reason the variable “feces management” was not included in this table? Presumably food animals would have access to feces that were left to disintegrate in the yard, for example.*

Feces management was included in Table 7. Table 6 represents variables that were considered household characteristics and risk factors. Table 7 represents variables that were considered household knowledge, attitudes, and practices. This is a consistent way in which the variables included in this study were divided into tables.

*L472: avoid contractions*

This has been revised.

*L603: define this acronym*

This acronym has been defined.

Reviewer #2:

*1. Grammatical Errors
There are a number of grammatical errors, which need to be corrected. Kindly go through the entire manuscript and correct them. For example:
Page 3, line 64-66: “However, third-generation cephalosporin resistant (3CGR) bacteria, including those than can produce extended-spectrum β-lactamases (ESBLs), are becoming increasingly common (2–5).”*
*Page 6, Line 116-120: “While risk factors for childhood colonization with ESBL E. coli in the region have been studied, specific household characteristics and caregiver antimicrobial knowledge, attitudes, and practices (KAP) that contribute to domestic animal colonization with 3GCR and ESBL E. coli have yet to be explored (63).”*

In addition to the changes outlined above, we have edited the paper thoroughly and made minor grammatical and clarity corrections. We have also edited all tables and included the revised versions in our new submission.

*2. From your statement below (Page 5 & 6, line 108-112), Is there a link between limited research and risk factors for antibiotic resistant E. coli carriage in domestic animals? What is the justification for such statement? See below

Page 5 & 6, line 108-112: “The limited research that exists regarding risk factors for AR E. coli carriage in animals in small-scale production settings suggests the specific risk factors contributing to domestic animal colonization with ESBL and other AR E. coli are likely context-dependent, warranting closer attention in specific settings where small-scale food animal production occurs (26,29,35).”*

This is a misunderstanding of the intent of this sentence. This statement is meant to express that there is limited research on this topic, but the research that does exist suggests risk factors are context-specific. This sentence has been corrected to try to avoid this alternative interpretation.

*3. Page 6, line 130-131: You stated that data was collected in three cycles. However, it is not clear what constitute a cycle. Clearly define what constitute a cycle.*

This has been clarified.

*4. Page 7, line137: What is the knowledge, attitudes, and practices (KAP) survey about? Is it about antibiotic usage? The statement should be completed.*

The word “antibiotic” has been inserted to complete this statement.

*5. Page 7, line138-139: You have stated that the KAP survey questionnaire used in this study has been previously validated. Kindly provide reference to that effect.*

Survey questions were derived from other KAP-based studies (see below), and questions were discussed and modified with local experts to ensure that the questions would be appropriate for the study’s specifications. Surveys were first developed in English, translated to Spanish, and then translated back to English. The interview team field-tested the questions to determine whether they were culturally appropriate and relevant. The surveys were adjusted based on feedback during the first four weeks of sampling. This information has been added to the manuscript under the “Knowledge, attitudes and practices (KAP) survey” subheading along with the following citations:

Mouhieddine TH, Olleik Z, Itani MM, Kawtharani S, Nassar H, Hassoun R, et al. Assessing the Lebanese population for their knowledge, attitudes and practices of antibiotic usage. J Infect Public Health. 2015;8(1):20–31.

Grigoryan L, Burgerhof JGM, Degener JE, Deschepper R, Lundborg CS, Monnet DL, et al. Attitudes, beliefs and knowledge concerning antibiotic use and self-medication: a comparative European study. Pharmacoepidemiol Drug Saf. 2007;16(11):1234–43.

*6. Page 10, 216: For consistency, use the terms “univariable and multivariable” instead of “univariate and multivariable”.*

This has been corrected.

*7. From the data you have presented in Table 1, cat is one of the common domestic animals you have sampled in all the three sample collection cycles. However, you collected only one faecal sample from a cat. What were the hindrances to collecting cat faecal samples and how is this likely to affect the interpretation of your results?*

The limitations portion of the “Discussion” has been updated to explain this consideration.

*8. You stated several times in your results section that you adjusted for confounders. However, you fail to mention the particular confounders you adjusted for in the text or table.*

Confounders have been included in Supplemental Table 1.

*9. Antimicrobial is not synonymous to antibiotics. Antimicrobials include antibiotics, antifungals, antivirals etc. Your statement above is therefore not correct. Modify the phrase “correctly answering no”.*

“Antimicrobials “has been updated to “antibiotics,” which is correct based on the wording used in the survey for this question. This is now consistent among all in-text and supplemental tables.

*10. You did not link Table 5 to the appropriate text. Kindly do that.*

Table 5 is now cited in the text.

*11. Dogs at households that obtained antimicrobials from a pet food store had increased odds of ESBL E. coli (OR 6.83, 95% CI 1.32-35.35). Conversely, a purchase of antimicrobials from a pet food store (OR 0.47, 95% CI 0.25-0.89) resulted in lower odds of food animal CR E. coli carriage. It is important to provide explanation for this disparity.*

Hypotheses for the explanation for this difference have been better outlined and discussed in the ‘Discussion” section of this paper.

*12. Page 27, line 432-434: What is the reason for comparing the prevalence of CR E. coli and ESBL E. coli carriage among domestic animals with human carriage at the first instance, when your study is about domestic animals carriage? Are there no similar studies among domestic animals you can compare to before comparing to human carriage?*

Similar studies among domestic animals have been included in the text of the discussion and compared to the results obtained in our study. This statement was meant to express the interesting phenomenon that our discovered prevalence is similar to that reported in the human population worldwide, which is relevant given our One Health focus. This sentence has been revised to clarify its purpose.

In addition to the changes discussed here, we have also added an additional author, Diana Paredes, who was inadvertently excluded from the original submission’s list of authorship. This error has now been corrected.
